# Valsartan Reduces Myocardial Ischemia–Reperfusion Injury by Inhibiting Ferritinophagy‐Mediated Ferroptosis

**DOI:** 10.1111/jcmm.71269

**Published:** 2026-07-02

**Authors:** Xumin Wang, Bixue Zhang, Xin Li, Qianqian Xu, Yuanliang Sun, Kaikai Zhao, Xintao Zhou, Yuhong Wu, Zheng Cheng

**Affiliations:** ^1^ Department of Cardiology Sixth Affiliated Hospital, Xinjiang Medical University Urumqi China; ^2^ Department of Otolaryngology Sixth Affiliated Hospital, Xinjiang Medical University Urumqi China; ^3^ Department of Basic Medical Science Academy Air Force Medical University Xi'an Shaanxi China; ^4^ Department of Respiratory and Critical Care Medicine Second Affiliated Hospital, Xi'an Jiaotong University Xi'an Shaanxi China; ^5^ Department of Cardiology Sinopharm Dongfeng General Hospital, Hubei University of Medicine Shiyan Hubei China; ^6^ Department of Cardiology Second Affiliated Hospital, Harbin Medical University Harbin Heilongjiang China; ^7^ Department of Cardiology Second Affiliated Hospital, Xi'an Jiaotong University Xi'an Shaanxi China

**Keywords:** ferritinophagy, ferroptosis, Mst1, myocardial ischemia–reperfusion injury, valsartan

## Abstract

Revascularisation is the key therapeutic strategy for acute myocardial infarction (AMI). However, the opening of the ‘culprit artery’ is accompanied by myocardial ischemia–reperfusion injury (IRI), partially offsetting the benefits of revascularisation. At present, effective methods to alleviate myocardial IRI are lacking. Cardiomyocyte ferritinophagy induces myocardial ferroptosis and IRI. In the present study, the RT‐PCR, western blotting, co‐immunoprecipitation, and immunofluorescence co‐localisation experiments revealed that hypoxia‐reoxygenation (H/R) of cardiomyocytes induces the dephosphorylation of Mst1 at Thr183, a crucial autophagy inhibitory site. Its dephosphorylation impairs the phosphorylation of Beclin1 at Ser295, thereby potentiating ferritinophagy, triggering ferroptosis and exacerbating H/R‐induced cardiomyocyte injury. In addition, valsartan restored the phosphorylation of Mst1 at Thr183 and Beclin1 at Ser295, thereby inhibiting myocardial ferritinophagy and ferroptosis. Valsartan also inhibits cardiomyocyte ferritinophagy by blocking the angiotensin II type 1 receptor. In vivo, valsartan ameliorated myocardial ferritinophagy, ferroptosis and IRI. However, following treatment with a ferroptosis inhibitor, valsartan failed to further alleviate myocardial IRI. In cardiomyocyte‐specific Mst1 knockout mice, valsartan failed to suppress myocardial ferritinophagy and ferroptosis, and consequently showed no therapeutic efficacy against IRI. Pre‐revascularisation administration of valsartan attenuated revascularisation‐induced myocardial injury and improved cardiac function in patients with AMI. These findings suggest that valsartan may mitigate myocardial IRI and improve clinical outcomes in patients with AMI by regulating the AT_1_R/Mst1/Beclin1 axis, thus inhibiting myocardial ferritinophagy and ferroptosis.

**Trial Registration:**

The Chinese Clinical Trial Registry (TRN ChiCTR2100043501)

## 1 Introduction

Coronary heart disease (CHD) is the most common cardiovascular disease in cardiology and has a substantial economic impact on patients [1]. Coronary heart disease arises from complex interactions among hereditary (genetic), environmental, and lifestyle factors [2]. Despite advances in pharmacotherapy, CHD remains a leading cause of morbidity and mortality worldwide [2]. For instance, in the United States alone, 1.2 million individuals experience a myocardial infarction annually, and approximately 40% of these events result in death [3]. Acute myocardial infarction (AMI), the most severe form of CHD, requires timely revascularisation [1]. However, this procedure simultaneously provokes a transient hemodynamic instability termed ‘myocardial ischemia‐reperfusion injury (IRI)’, which manifests as hypotension, malignant arrhythmias or sudden cardiac death during the reperfusion phase, consequently resulting in compromised cardiac function and partially offsetting the benefits of revascularisation [4]. At present, no drugs have proven effective at reducing myocardial IRI, highlighting the critical need to elucidate its mechanisms and develop appropriate therapies [5].

Ferroptosis, a form of cell death that is iron‐dependent and non‐apoptotic, is characterised by the accumulation of cytosolic labile Fe^2+^ and an overload of lipid reactive oxygen species (ROS)/peroxides [6, 7]. Previous studies corroborate that cardiomyocyte ferroptosis is exacerbated during reperfusion and that its inhibition can mitigate cardiomyocyte injury [6, 8, 9].

Abnormal iron metabolism and excessive lipid peroxidation are the triggers of ferroptosis [10]. Thus, agents such as iron chelators, antioxidants, ACSL4 inhibitors, nitric oxides, and lipoxygenase inhibitors may inhibit myocardial ferroptosis [10]. However, these agents are inefficient in treating ferroptosis‐induced myocardial IRI due to severe toxic side effects and difficulty in achieving safe plasma concentration [11, 12]. Therefore, it is imperative to develop innovative therapies for the treatment of myocardial ferroptosis and IRI.

Autophagy, an evolutionarily conserved process, functions to eliminate damaged organelles and proteins to maintain cellular homeostasis [13]. Physiological autophagy facilitates cellular repair and metabolic processes. However, excessive autophagy culminates in autophagic cell death [13, 14, 15]. Ferroptosis is a form of autophagy‐dependent cell death [7]. In myocardial IRI, the increase in autophagy facilitates ferroptosis through the degradation of ferritin, a process termed ferritinophagy [16, 17]. NCOA4, a selective receptor for ferritinophagy, directs ferritin to autophagosomes for subsequent degradation [18, 19]. Accordingly, targeted inhibition of the ferritinophagy receptor may serve as one therapeutic strategy against myocardial ferroptosis and IRI [18, 19].

Mst1, an important regulator of the Hippo/YAP pathway, is universally expressed in human organs and performs vital functions in promoting cell adhesion and migration while inhibiting autophagy [20]. Our previous studies demonstrated that the phosphorylation of Mst1 at Thr183 suppresses myocardial autophagy [14, 21, 22]. However, the involvement of Mst1 Thr183 phosphorylation in myocardial ferritinophagy and ferroptosis has not yet been elucidated [14, 21]. Previous findings also indicate that the angiotensin II type 1 receptor (AT_1_R) may augment myocardial autophagic flux, a process potentially modulated by Mst1 and valsartan [14]. Nevertheless, the roles of the valsartan‐AT_1_R‐Mst1 axis in myocardial ferritinophagy, ferroptosis, and IRI have not yet been elucidated.

The objectives of the present study included: (i) establishment of an in vitro cardiomyocyte H/R model and an in vivo myocardial IRI model with the purpose of elucidating the regulatory effects of the AT_1_R‐Mst1 pathway on ferritinophagy and ferroptosis; and (ii) determining whether valsartan ameliorates myocardial IRI by regulating the Mst1 pathway.

## 2 Materials and Methods

### 2.1 Participants and Data Collection

The present study was registered at the Chinese Clinical Trial Registry (https://www.chictr.org.cn/) and received approval from The Medical Ethics Committee of Sinopharm Dongfeng General Hospital in Shiyan City of China (approval no. 2020016). A total of 32 patients with AMI undergoing emergency revascularisation were enrolled and randomly assigned 1:1 to the valsartan pretreatment group (*n* = 16) and the conventional treatment group (*n* = 16) using block randomisation. All patient data were anonymised and confidential.

### 2.2 Animal Model Construction

The experimental animals were male, had a C57BL/6J background and were marked with ear tags (preliminary experiments, *n* = 10; formal experiments, *n* = 40). Mst1 conditional knockout mice on a C57BL/6 background were purchased from Cyagen Biosciences Inc. (contract no. GTC161121CAP01). Mst1^flox/flox^ mice were bred with αMHC‐MerCreMer mice to generate F3 offspring (Mst1^flox/+^: αMHC‐MerCreMer). Subsequent backcrossing of F3 mice with Mst1^flox/flox^ mice produced the F4 progeny, Mst1^flox/flox^: αMHC‐MerCreMer mice, as previously described [14, 21, 22].

The animal experimental protocol was reviewed and approved by The Experimental Animal Ethics Committee of Xinjiang Medical University in Urumqi City of China (approval no. IACUC‐20250306‐19), and complies with NIH guidelines and the Regulations on the Administration of Laboratory Animals [23]. All animals were raised in The Animal Experiment Center of Xinjiang Medical University in Urumqi City of China, and feeding was completed by the project researchers.

Prior to surgery, valsartan (4 mg/kg) was administered via intragastric gavage. The myocardial ischemia–reperfusion (I/R) model was established via thoracotomy and ligation of the left anterior descending coronary artery. All surgical procedures were performed under anaesthesia using isoflurane (induction, 4.0%; maintenance, 1.5%, HE BEI YI PIN Pharmaceutical Co. Ltd) and buprenorphine (subcutaneous injection, 0.1 mg/kg) was used for preoperative analgesia; the pain level was staged as Class B (moderate pain) [24].

For euthanasia, animals were euthanised by intraperitoneal injection of sodium pentobarbital (100 mg/kg) followed by cervical dislocation. All experimental samples were collected after the animals were sacrificed.

### 2.3 Plasmid and Adenovirus Construction

Adenoviruses carrying Mst1 shRNA (Ad‐Mst1 shRNA) were obtained from Hanbio Biotechnology Co. Ltd. [14, 21, 22]. The expression vector pHBAd‐U6‐Scramble‐CMV was engineered using EcoR I and BamH I restriction sites. Viral titers were quantified as follows: Ad‐Mst1 shRNA1 (1 × 10^10^ PFU/mL), Ad‐Mst1 shRNA2 (1.26 × 10^10^ PFU/mL) and Ad‐Mst1 shRNA3 (1.58 × 10^10^ PFU/mL). The effectiveness of Ad‐Mst1 shRNA was verified via immunofluorescence (IF) and western blotting (WB), and Ad‐Mst1 shRNA2 was selected as the optimal adenovirus. pcDNA3.1‐AT_1_R was used to transfect 293T cells, with pcDNA3.1‐EGF acting as the control (Xi'an Zhuangzhi Biotechnology Co. Ltd). Finally, positive clones were selected and verified using RT‐PCR and WB.

### 2.4 Isolation, Culture and Identification of Neonatal Mouse Primary Cardiomyocytes

Primary ventricular cardiomyocytes were isolated from wild‐type C57BL/6J neonatal mice (age, 1–3 days), as previously described [14, 21, 22].

### 2.5 Culture of H9C2 Cells

H9C2 cells were purchased from Xi'an Sxfcbio Co. Ltd. (cat. no. FC‐cell‐181121A).

### 2.6 Cell Viability Analysis

MTT assays were implemented to detect cell viability according to standard instructions (98% Reagent grade, Biotechnology, cat. no. ST1537).

### 2.7 Adenoviral Transduction and GFP‐LC3/GFP‐mRFP‐LC3 Detection

The GFP‐LC3 and GFP‐mRFP‐LC3 adenoviruses were obtained from Hanbio Biotechnology Co. Ltd. [14]. Transduction procedures were conducted in accordance with established methods [14]. In cardiomyocytes expressing GFP‐mRFP‐LC3, LC3 associated with autophagosomes is visualised as yellow puncta, whereas autolysosomes are visualised as red puncta [14].

### 2.8 Echocardiograph

Before the mice were sacrificed, cardiac function was assessed pre‐euthanasia using a Vevo 2100 echocardiography system (VisualSonics Inc.) with a 15‐MHz linear transducer [14, 21]. M‐mode imaging was performed to evaluate left ventricular parameters [14, 21].

### 2.9 Transmission Electron Microscopy (TEM)

The sediment of cardiomyocytes was put into a centrifuge tube containing glutaraldehyde for 2 h [14]. After which, the precipitates underwent permeation, dehydration, and embedding overnight at 4°C [14]. These samples were post‐stained with 5% uranyl acetate and 0.2% lead citrate and finally viewed using a transmission electron microscope (Jeol JEM‐1200EX TEM; 60 kV) to assess ultrastructure, specifically, the mitochondrial ultrastructure.

### 2.10 Mitochondrial Membrane Potential (ΔΨm) Assessment

ΔΨm was measured using JC‐1 dye (Beyotime Biotechnology, cat. no. C2006) according to manufacturer's guidelines.

### 2.11 SYTOX Green Stains

The mortality of cardiomyocytes was assessed using SYTOX Green Nucleic acid dye (Ex/Em, 502/525 nm; bound to DNA) according to the manufacturer's instructions. The nucleus of dead cells exhibits green fluorescence. In vivo, paraffin‐embedded myocardial sections (thickness, 6 μm) underwent deparaffinisation, and five random sections/heart were selected for analysis.

### 2.12 Histological Analysis

Formalin‐fixed heart specimens were paraffin‐embedded, sectioned (thickness, 6 μm) and stained with Masson's trichrome and Sirius red for fibrosis evaluation [14, 21]. The Evans Blue/TTC stains of myocardium were conducted in accordance with established methods [25].

### 2.13 Immunohistochemistry (IHC)

Antigen retrieval was performed via microwave treatment on paraffin sections [14, 21]. Primary antibodies (1:200) were incubated overnight, with isotype IgG serving as the negative control [14, 21]. Signal development used 3,3′‐diaminobenzidine, and quantification averaged 8–10 fields/section [14, 21].

### 2.14 Dual Labeling of Immunofluorescence Co‐Localisation (IFC)

IFC of GFP‐LC3/NCOA4, GFP‐LC3/FTH1, Beclin1/ATG14, and NCOA4/FTH1 utilised sequential staining protocols [14, 21].

### 2.15 WB and Real‐Time PCR

Total RNA/protein was extracted from left ventricles or primary cardiomyocytes. qPCR was performed using a Bio‐Rad CFX96 system, and protein quantification employed the Bradford assay (Bio‐Rad Laboratories Inc.). The blots were visualised using a UVP chemiluminescence system [14, 21, 22].

### 2.16 Co‐Immunoprecipitation (Co‐IP)

The protein interactions of Mst1‐Beclin1, Beclin1‐Bcl‐2, and NCOA4‐FTH1 were investigated using the Dynabeads (Millipore, PureProteome Protein A and Protein G Magnetic Beads, cat. no. LSKMAGAG10) according to the methodologies described in the literature [21, 22].

### 2.17 Antioxidant Measurement, Fe^2+^ Release and Lipid Peroxidant Assessment

The antioxidant (GPx4, GSH, and NAPDH) levels were quantified using assay kits, respectively. Fe^2+^ release assay was measured by the Iron Assay Kit (cat. no. K390‐100; AmyJet Scientific Inc.). Lipid ROS, LPO, MDA, and NAPDH oxidase levels were tested using commercialised assay kits, respectively.

### 2.18 Serum Biomarkers of Myocardial Damage

The levels of serum creatine kinase‐MB (CK‐MB), lactate dehydrogenase (LDH), aspartate aminotransferase (AST), cardiac troponin T (cTNT), and brain natriuretic peptide (BNP) were measured by commercialised assay kits, as described in the protocol.

### 2.19 DHE Probe Detection

Ventricular tissue was frozen using OCT embedding compound (Sakura Finetek Europe B.V.; cat. no. 4583) and sectioned into 8 μm cryosections using a cryostat (Thermo Fisher Scientific Inc.; Cryotome E). A circle was drawn around the myocardial tissue to minimise reagent waste, and within the circle, the sections were incubated with the fluorescent probe dihydroethidium (DHE; 5 mmol/L; Beyotime Biotechnology, Beijing; cat. no. S0063) at 37°C for 45 min in the dark. Subsequently, 2–3 drops of DAPI staining solution were added, and the sections were incubated for ~11 min at room temperature in the dark. After which, the sections were mounted with an anti‐fade mounting medium. Fluorescence images were captured and analysed using a confocal laser scanning microscope (Olympus FV100; Olympus Corporation) to assess DHE fluorescence intensity in the myocardial tissue, which reflects the level of superoxide in situ [21].

### 2.20 Statistical Analysis

The data are presented as the mean ± SEM. For quantitative data, two groups were compared using one‐way ANOVA. *p* < 0.05 was considered to indicate a statistically significant difference. Two‐sided tests were used throughout the present study.

Survival was estimated by the Kaplan–meier method, and any differences in survival were evaluated with a stratified log‐rank test. The analyses were conducted utilizing GraphPad Prism (version 5.01; Dotmatics) and SPSS (version 27; IBM, Corp.).

## 3 Results

### 3.1 Autophagy and Ferroptosis Were Associated With Myocardial IRI and May Involve Mst1 and Ang II

**FIGURE 1:**
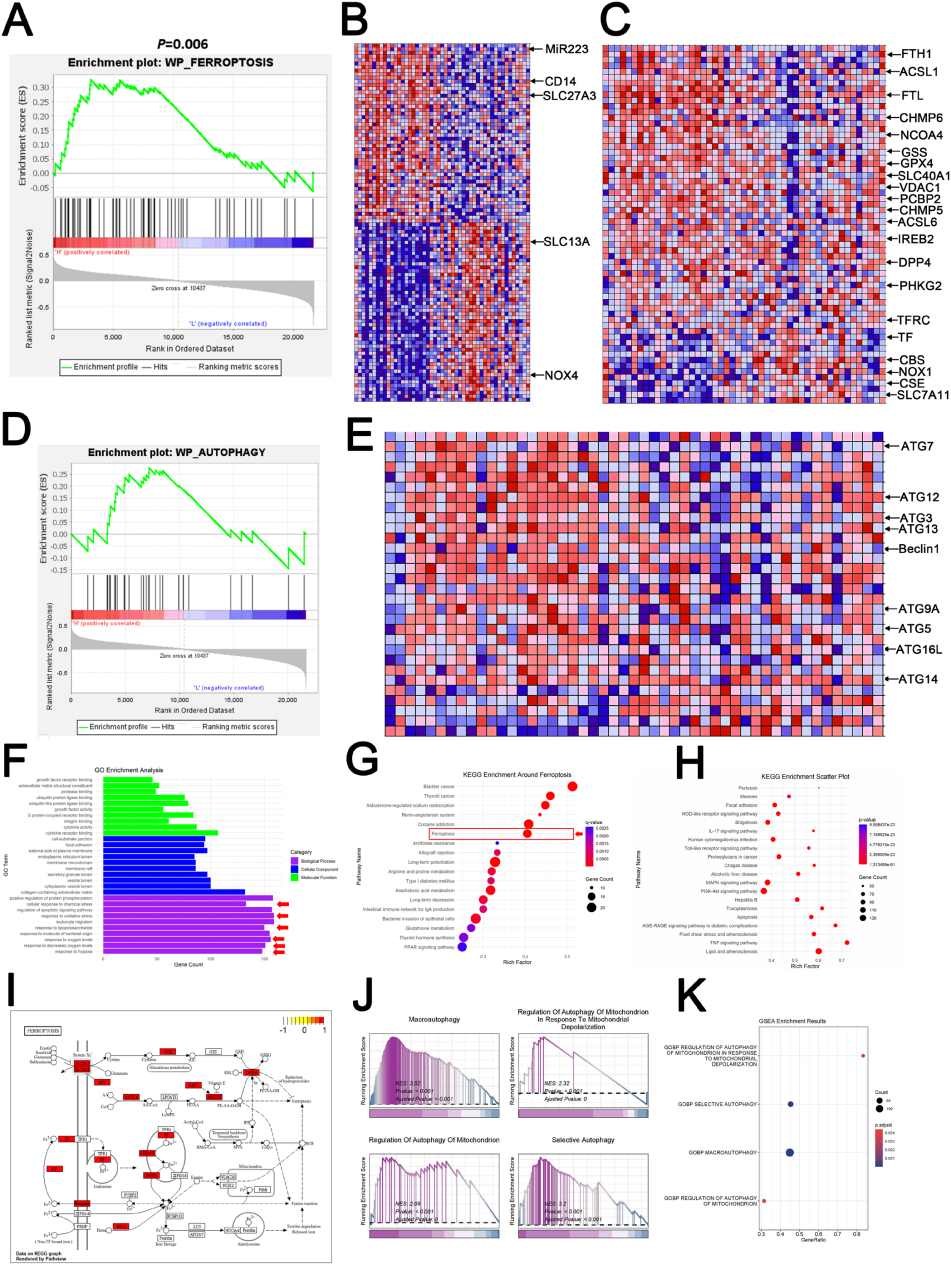
Autophagy and ferroptosis are associated with myocardial IRI and may involve Mst1 and Ang II. (A–C) The analyses of GESA (GSE66360): The enrichment plot of ferroptosis‐related pathways and the heat map of molecules with differences. (D, E) The analyses of GESA (GSE66360): The enrichment plot of autophagy‐related pathways and the heat map of autophagy‐related molecules. Beclin1 is the leading‐edge subset, with Rank Metric Score = 0.119. (F) The Go Enrichment Analysis: The Go terms closely related to ferroptosis in myocardial IRI. (G) The KEGG Enrichment Analysis: The ferroptosis‐related pathway is the crucial pathway involved in myocardial IRI. (H) The KEGG Enrichment Scatter Plot: Among the pathways associated with AMI and IRI, Mst1 (Entrez 6789) was enriched in essential KEGG pathways. (I) Diagram: Genes related to ferritinophagy and ferroptosis were prominently enriched in the myocardial I/R dataset. (J, K) The analyses of GESA (GSE43974, T3 = 60 min after I/R): The enrichment plot and GESA results of pathways and biological function.

By employing the GSE66360 dataset as a test cohort, gene set enrichment analysis (GSEA) demonstrated that pathways related to ferroptosis were significantly upregulated in the AMI group compared with those in the control group (Figure 1A). The GSEA heat maps are comprehensively illustrated in Figure 1B,C. Alterations in autophagy pathways induced by AMI were identified, accompanied by aberrant upregulation of autophagy‐related genes. Beclin1, a crucial regulator of autophagy, was recognised as the leading‐edge subset within the core enrichment (Figure 1D,E).

Gene Ontology enrichment analysis revealed the following GO terms that were strongly associated with myocardial IRI and ferroptosis: ‘the response to oxidative stresses’ (a primary initiator of ferroptosis), ‘the cellular response to chemical stress’ (associated with iron accumulation and increased ROS levels) and ‘the response to H/R’ (essential for the activation of ferroptosis; Figure 1F). KEGG pathway analysis further validated ferroptosis as one of pivotal pathways involved in IRI (Figure 1G). Among the pathways associated with AMI and IRI, Mst1 (Entrez Gene ID 6789) was enriched in essential KEGG pathways, such as hsa04010 MAPK signalling, hsa04068 FoxO signalling, and hsa04014 Ras signalling (Figure 1H). Valsartan is involved in myocardial oxidative stress and ferroptosis via the MAPK/Mst1, FoxO/Mst1, and NOX/ROS pathways [14, 26, 27]. Genes related to ferritinophagy and ferroptosis were prominently enriched in the myocardial IR dataset (https://www.kegg.jp/pathway/hsa04216; Figure 1I).

Enrichment analysis of the GSE43974 dataset involving Mst1 revealed significant enrichment in autophagosome/autolysosome‐related pathways, including macroautophagy, mitophagy, and selective autophagy, such as ferritinophagy (Figures 1J,K and S1A,B). Spearman correlation analysis between Mst1 and other genes was performed. Mst1 expression was positively correlated with the expression of Beclin1 (*p* < 0.001), AT_1_R (*p* < 0.001), and RND3 (*p* < 0.001; Figure S1C–E).

### 3.2 H/R Induced Cardiomyocyte Ferroptosis Accompanied by Mst1 Dephosphorylation at Thr183 and Ferritinophagy Overactivation

**FIGURE 2:**
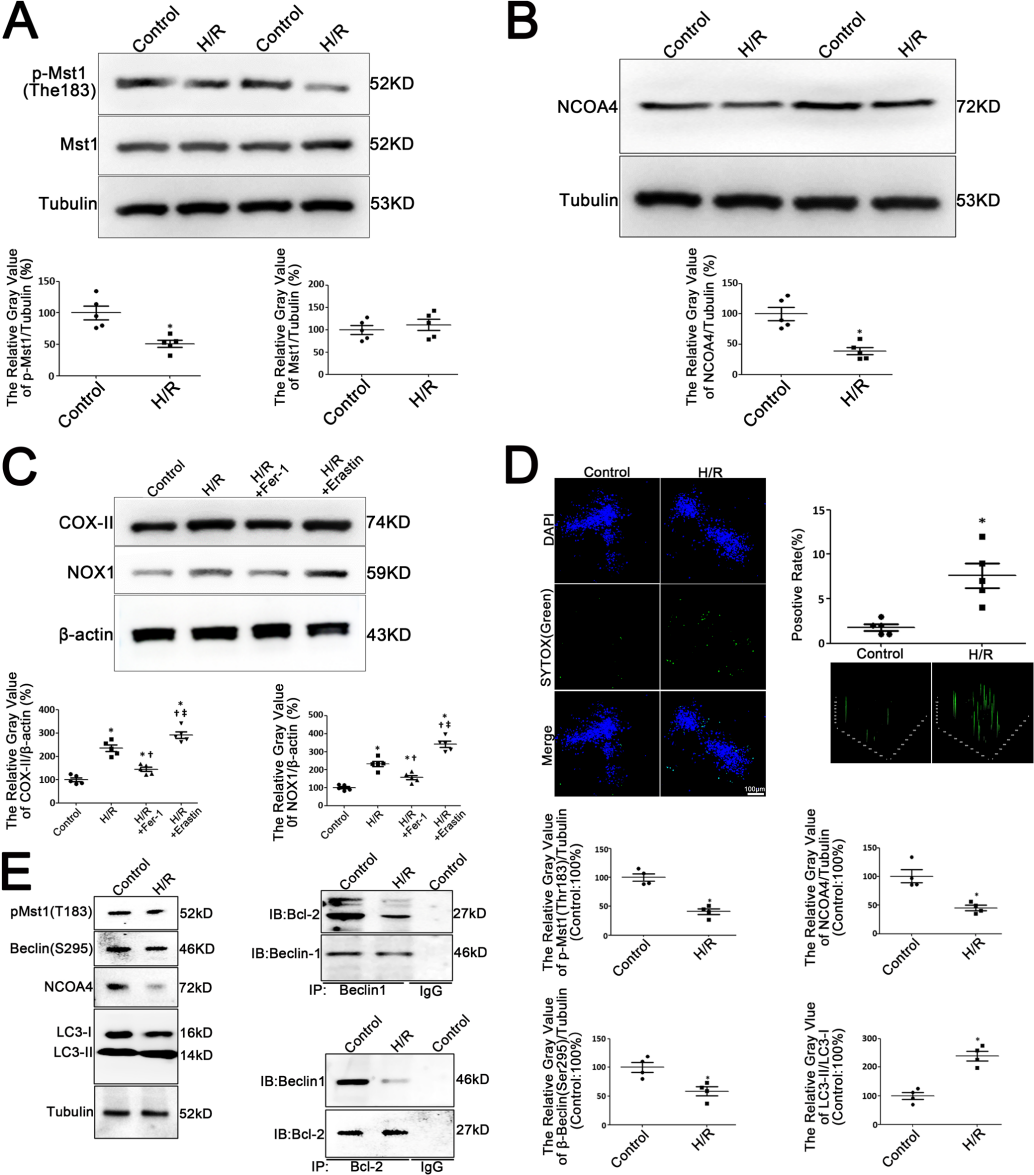
H/R induces cardiomyocyte ferroptosis accompanied by Mst1 dephosphorylation at Thr183 and ferritinophagy overactivation. (A–C) Immunoblots and quantitative analyses of *p*‐Mst1 (Thr183)/Tubulin, Mst1/Tubulin, NCOA4/Tubulin, COX‐II/β‐actin and NOX1/β‐actin. Histogram: The Relative Grey Value of *p*‐Mst1 (Thr183)/Tubulin, Mst1/Tubulin, NCOA4/Tubulin, COX‐II/β‐actin and NOX1/β‐actin. *p < 0.05 vs. Control group; ^†^ *p* < 0.05 vs. H/R group; ^‡^ *p* < 0.05 vs. H/R + Fer‐1, (*n* = 5). (D) Representative immunofluorescence images of SYTOX (Green). Histogram: The positive rate of cardiomyocytes (Death Rate %) *p < 0.05 vs. Control group, (*n* = 5). (E) The representative images of Immunoblots and Co‐IP (IP: Beclin; IP: Bcl‐2). Histogram: The Relative Grey Value of *p*‐Mst1 (Thr183)/Tubulin, Beclin1 (Ser295)/Tubulin, NCOA4/Tubulin, and LC3‐II/LC3‐I. *p < 0.05 vs. Control group, (*n* = 4).

In cardiomyocytes, WB revealed that H/R treatment induced the dephosphorylation of Mst1 at Thr183 and decreased the expression of NCOA4 (Figure 2A,B). However, the relative mRNA levels of NCOA4 and FTH1 were unchanged (Figure S2A,B). Notably, following the treatment of H/R, the increased expression of COX‐II and NOX1, markers of lipid ROS, lipid peroxides and ferroptosis, were reversed by a ferroptosis inhibitor or further elevated by a ferroptosis inducer (Figure 2C). H/R caused an increase in lipid ROS, LDH, lipid peroxidation and Fe^2+^ release (Figure S2C–H). Moreover, H/R induced the loss of antioxidant capacity and the deletion of ROS‐scavenging enzymes, including GSH, GPX4 and NAPDH (Figure S2I–K). SYTOX Green nucleic acid staining revealed that H/R decreased the viability of cardiomyocytes (Figure 2D).

Co‐IP revealed that H/R weakened the interaction between Beclin1 and Bcl‐2, dephosphorylated Mst1 at Thr183, an inhibition site of autophagy, and decreased Beclin1 phosphorylation at Ser295. WB assays showed an increase in LC3‐II/LC3‐I and a decrease in the ferritinophagy receptor NCOA4 (Figure 2E).

### 3.3 Valsartan Attenuated H/R‐Induced Cardiomyocyte Ferroptosis and Ferritinophagy by Phosphorylating Mst1 at Thr183

**FIGURE 3:**
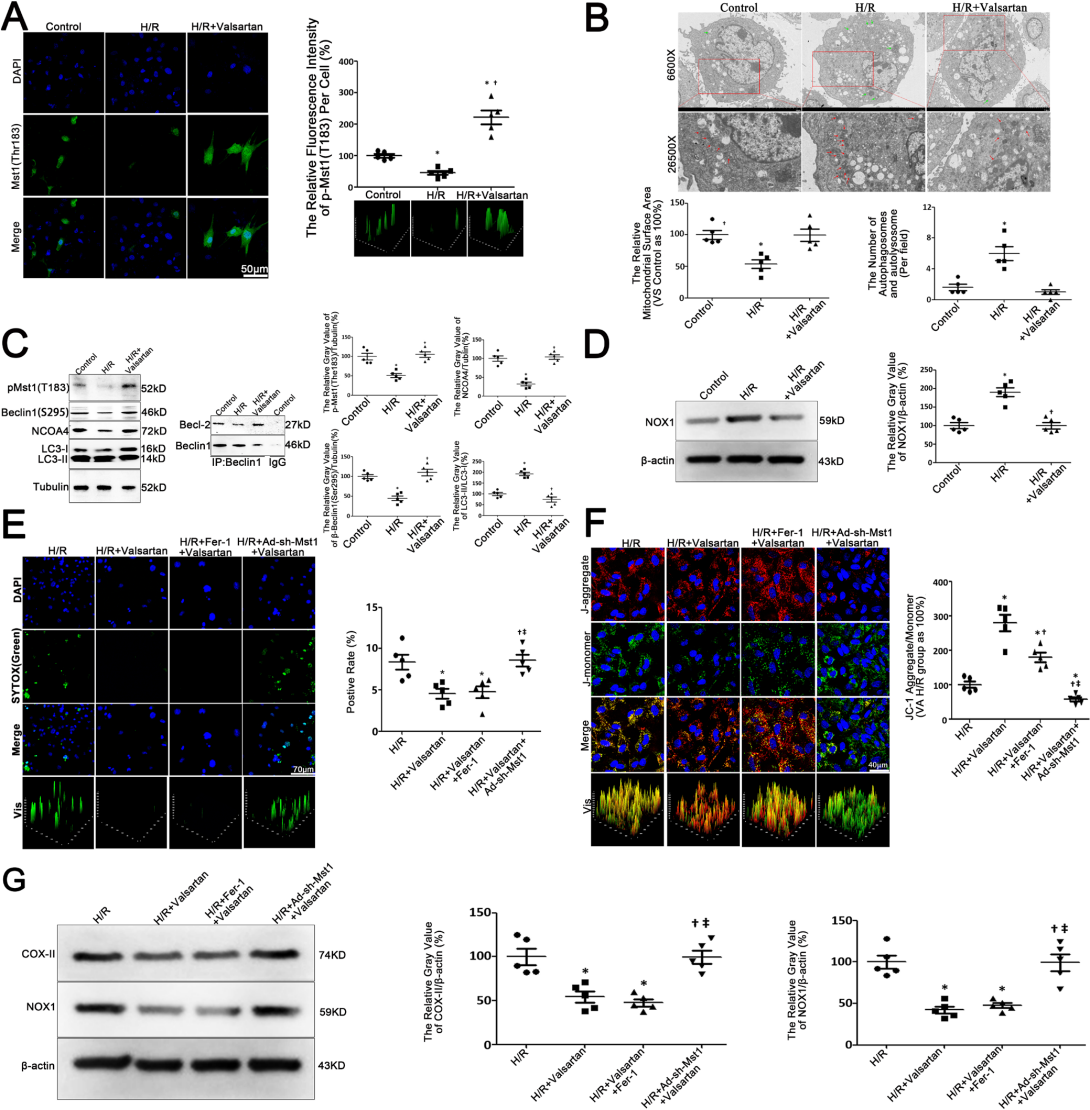
Valsartan attenuates H/R‐induced cardiomyocyte ferroptosis and ferritinophagy by phosphorylating Mst1 at Thr183. (A) Representative immunofluorescence images of Mst1 (Thr183); Histogram: The relative fluorescence intensity of Mst1 (Thr183). Control group as 100%. *p < 0.05 vs. Control group, ^†^ *p* < 0.05 vs. H/R group (*n* = 5). (B) Representative TEM images of cardiomyocyte. Histogram: The number of autophagosomes and autolysosomes; the relative mitochondrial surface area. Control group as 100%. *p < 0.05 vs. Control group, ^†^ *p* < 0.05 vs. H/R group (*n* = 5). (C) The representative images of Immunoblots and Co‐IP (IP: Beclin). Histogram: The Relative Grey Value of *p*‐Mst1 (Thr183)/Tubulin, Beclin1 (Ser295)/Tubulin, NCOA4/Tubulin, and LC3‐II/LC3‐I. *p < 0.05 vs. Control group, ^†^ *p* < 0.05 vs. H/R group (*n* = 5). (D) Immunoblots and quantitative analyses of NOX1/β‐actin. Histogram: The Relative Grey Value of NOX1/β‐actin. *p < 0.05 vs. Control group; ^†^ *p* < 0.05 vs. H/R group (*n* = 5). (E, F) Representative immunofluorescence images of SYTOX (Green) and JC‐1 stains. Histogram: The positive rate of cardiomyocytes (Death Rate %); The JC‐1 aggregate/monomer (H/R group as 100%), *p < 0.05 vs. H/R group; ^†^ *p* < 0.05 vs. H/R + valsartan group; ^‡^ *p* < 0.05 vs. H/R + Fer‐1 + valsartan group (*n* = 5). (G) Immunoblots and quantitative analyses of COX‐II/β‐actin and NOX1/β‐actin. Histogram: The Relative Grey Value of COX‐II/β‐actin and NOX1/β‐actin. *p < 0.05 vs. H/R group; ^†^ *p* < 0.05 vs. H/R + valsartan group; ^‡^ *p* < 0.05 vs. H/R + Fer‐1 + valsartan group (*n* = 5).

Valsartan improved the H/R‐induced dephosphorylation of Mst1 at Thr183 (Figure 3A). TEM analysis of cardiomyocyte ultrastructure revealed that H/R caused mitochondrial shrinkage, increased mitochondrial membrane density, loss or fragmentation of cristae, and outer membrane rupture, whereas the nuclear morphology remained intact (Figure 3B). These ultrastructural alterations are characteristic of ferroptosis. Notably, valsartan reversed H/R‐induced mitochondrial morphological abnormalities (Figure 3B).

Co‐IP and immunoblotting assays demonstrated that valsartan reversed the H/R‐induced protein–protein interactions and alterations of protein expression (Figure 3C). However, during this process, the mRNA levels of NCOA4 and FTH‐1 remain unchanged (Figure S3A,B). Valsartan failed to disrupt mitophagy in cardiomyocytes (Figure S3C–E). WB assays revealed that valsartan reversed the H/R‐induced increases in the expression levels of NOX1, a marker of ferroptosis (Figure 3D). SYTOX Green nucleic acid and JC‐1 staining revealed that valsartan alleviated H/R‐induced cardiomyocyte death and mitochondrial injury (Figure 3E,F). It can be observed that valsartan improved cardiomyocyte viability (Figure S3F). Moreover, valsartan reversed the H/R‐induced increases in factors that induce ferroptosis, such as lipid ROS, lipid peroxidation and Fe^2+^ release (Figure S3G–I). Valsartan also improved the H/R‐induced decreases in antioxidant molecules and ROS scavengers, such as GPX4, GSH and NAPDH (Figure S3J–L).

Notably, following treatment with valsartan, Fer‐1, an inhibitor of ferroptosis, failed to further alleviate the H/R‐induced cardiomyocyte death, mitochondrial injury, and increase in ferroptosis markers (Figure 3E–G). Additionally, when Mst1 was knocked out in cardiomyocytes, the protective effect of valsartan on H/R‐induced injury (HRI) disappeared (Figure 3E–G).

### 3.4 Valsartan Attenuated H/R‐Induced Cardiomyocyte Ferroptosis by Phosphorylating Mst1 to Regulate Ferritinophagy

**FIGURE 4:**
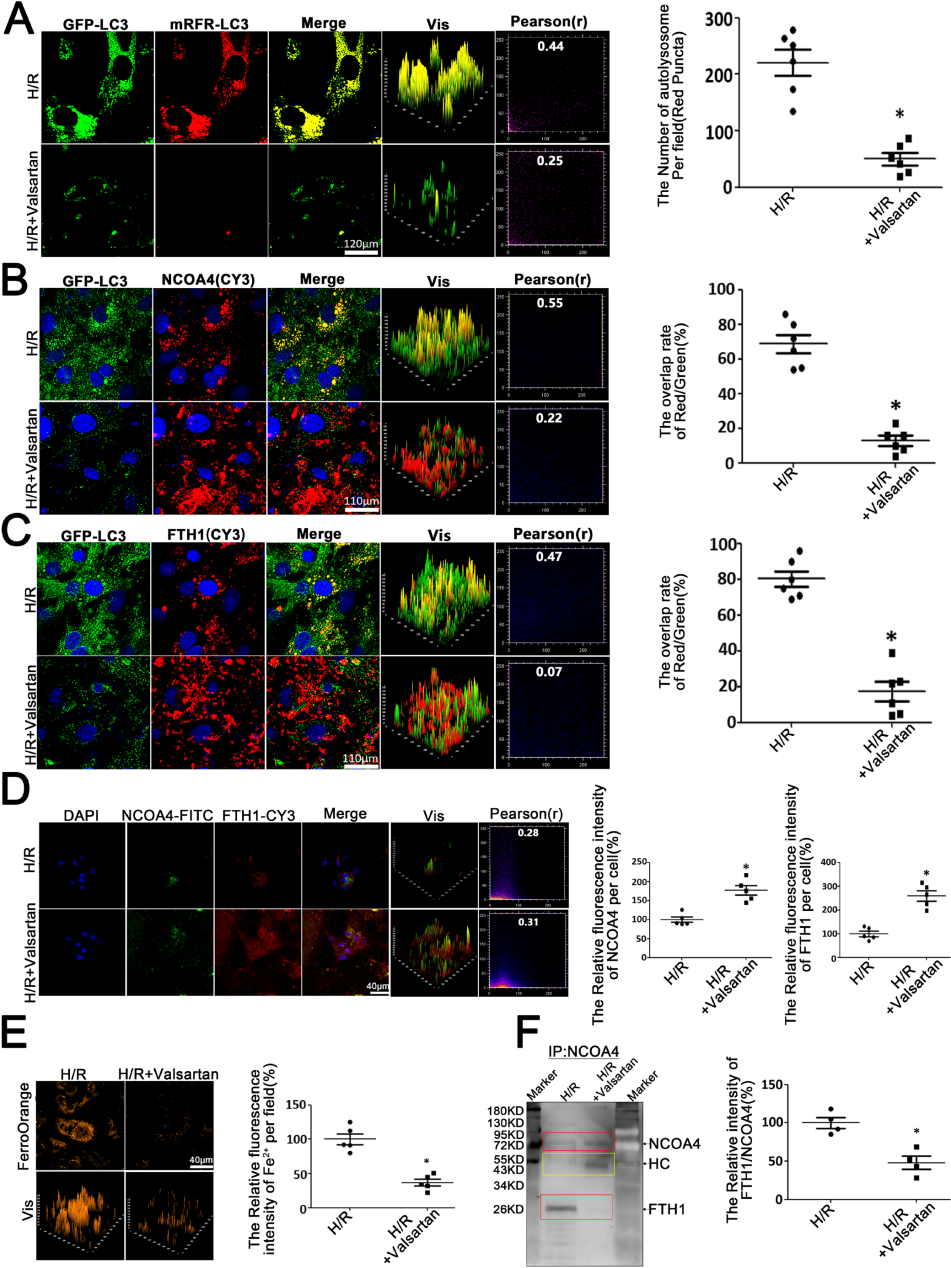
Valsartan attenuates H/R‐induced cardiomyocytes ferroptosis by phosphorylating Mst1 to regulate ferritinophagy. (A) Representative immunofluorescence images of GFP‐mRFP‐LC3 and Correlation analysis between GFP‐LC3 and mRFP‐LC3; Histogram: The number of autolysosomes per field (Red puncta per field). *p < 0.05 vs. H/R group, *n* = 6. (B–D) Representative images of immunofluorescence co‐localisation between GFP‐LC3 and NCOA4 (CY3); GFP‐LC3 and FTH1 (CY3); NCOA4 (FITC) and FTH1 (CY3). Histogram: The overlap rate of Red/Green (%), *n* = 6; the relative fluorescence intensity of NCOA4 per cell and the relative fluorescence intensity of FTH1 per cell (H/R group as 100%), *n* = 5. *p < 0.05 vs. H/R group. (E) Representative immunofluorescence images of FerrOrange; Histogram: The relative fluorescence intensity of free Iron ion per field (H/R group as 100%), *p < 0.05 vs. H/R group, *n* = 5. (F) The representative images of Co‐IP (IP: NCOA4). Histogram: The relative intensity of FTH‐1/NCOA4 (H/R group as 100%). *p < 0.05 vs. Control group, (*n* = 4).

IFC experiments revealed that valsartan inhibited autophagic flux and suppressed the recruitment of NCOA4 and ferritin to autophagosomes and autolysosomes in cardiomyocytes (Figures 4A–C and S4A,B).

Valsartan reversed the H/R‐induced decrease in NCOA4 and ferritin expression (Figures 4D and S4A–F). However, during this process, the mRNA levels of NCOA4 and ferritin remained unchanged, suggesting that valsartan inhibited the H/R‐induced degradation of NCOA4 and ferritin via the autophagy‐lysosome pathways (Figure S4G,H).

IF staining revealed that compared with the H/R group, the valsartan + H/R group had decreased free iron ions in the cytoplasm of cardiomyocytes (Figure 4E). Moreover Co‐IP revealed a stronger interaction between NCOA4 and FTH1 in the H/R + valsartan group compared with the H/R group (Figure 4F).

**FIGURE 5:**
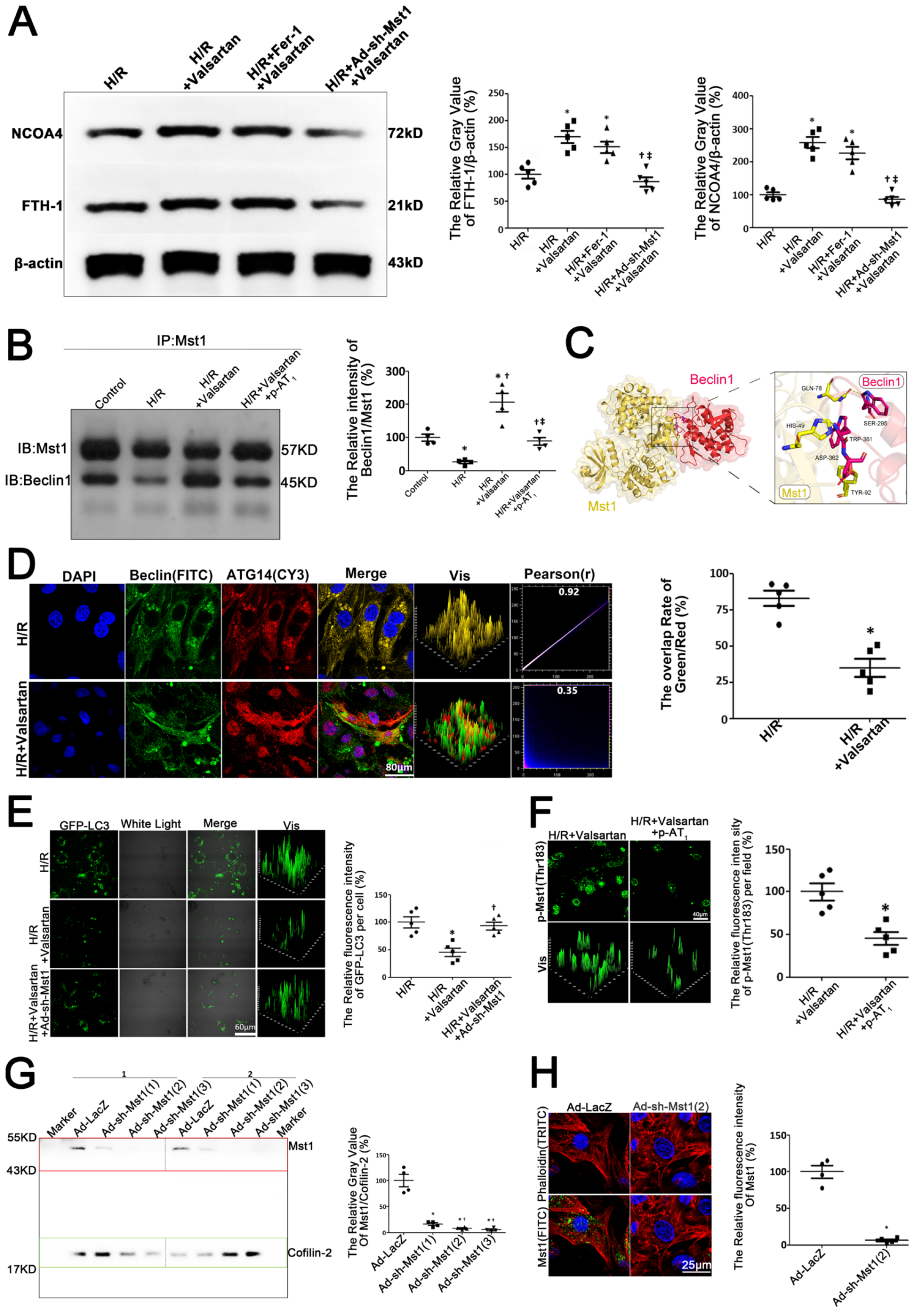
Valsartan attenuates H/R‐induced cardiomyocytes ferroptosis by phosphorylating Mst1 to regulate ferritinophagy. (A) Immunoblots and quantitative analyses of FTH‐1/β‐actin and NCOA4/β‐actin. Histogram: The relative Grey Value of FTH‐1/β‐actin and NCOA4/β‐actin. H/R group as 100%. *p < 0.05 vs. H/R group; ^†^ *p* < 0.05 vs. H/R + valsartan group; ^‡^ *p* < 0.05 vs. H/R + Ad‐sh‐Mst1 + valsartan group (*n* = 5). (B) The representative images of Immunoblots and Co‐IP (IP: Mst1). Histogram: The relative intensity of Beclin1/Mst1 (%). Control group as 100%. *p < 0.05 vs. Control group, ^†^ *p* < 0.05 vs. H/R group; ^‡^ *p* < 0.05 vs. H/R + valsartan group (*n* = 4). (C) The molecular docking between Mst1 and Beclin1. (D) Representative images of immunofluorescence co‐localisation between Beclin1 (FITC) and ATG14 (CY3). Histogram: The overlap rate of Green/Red (%), *n* = 5. *p < 0.05 vs. H/R group. (E, F) Representative immunofluorescence images of GFP‐LC3 and *p*‐Mst1 (Thr183); Histogram: The relative fluorescence intensity of GFP‐LC3 per field (H/R group as 100%), *p < 0.05 vs. H/R group; ^†^ *p* < 0.05 vs. H/R + valsartan group; *n* = 5. The relative fluorescence intensity of *p*‐Mst1 (Thr183) per field (H/R + valsartan group as 100%). *p < 0.05 vs. H/R + valsartan group; *n* = 5. (G) The representative images of Immunoblots. Histogram: The relative grey value of Mst1/Cofilin‐2 (%). Ad‐LacZ group as 100%. *p < 0.05 vs. Ad‐LacZ group, ^†^ *p* < 0.05 vs. Ad‐sh‐Mst1(1) group (*n* = 4). (H) Representative immunofluorescence images of Mst1 (FITC) and Phalloidin (TRITC); Histogram: The relative fluorescence intensity of Mst1 (FITC) per field (Ad‐LacZ group as 100%), *p < 0.05 vs. Ad‐LacZ group, *n* = 4.

WB experiments revealed that valsartan can increase NCOA4 and FTH‐1 expression following H/R (Figure 5A). Notably, the ferroptosis inhibitor Fer‐1 failed to further increase the expression of NCOA4 and FTH1 during treatment with valsartan (Figure 5A). Mst1 knockout abolished the valsartan‐induced increase in NCOA4 and ferritin expression (Figure 5A). However, the mRNA levels of NCOA4 and FTH‐1 remained unchanged (Figure S5A,B).

Co‐IP revealed stronger interaction between Mst1 and Beclin1 in the H/R + valsartan group than in the H/R group (Figure 5B). The effect of valsartan on the interaction between Mst1 and Beclin1 was weakened by overexpression of the AT_1_R (Figure 5B). Molecular docking suggested potential interaction sites between Mst1 and Beclin1, including Beclin1 at Ser295, an autophagy‐inhibitory site (Figure 5C). IFC assays revealed that following H/R, valsartan weakened the co‐localisation between Beclin1 and ATG14, suggesting inhibition of the autophagy initiation complex (Figure 5D). IF assays revealed that valsartan decreased the expression of LC3 following H/R, which was abolished by Mst1 inhibition (Figure 5E). IF experiments also revealed that valsartan phosphorylates Mst1 at Thr183, whereas overexpressing AT_1_R partially inhibited the Mst1 phosphorylation by valsartan (Figure 5F). The effectiveness of Mst1 knockdown in vitro was verified by the WB and IF assays (Figure 5G,H).

Following H/R, valsartan improved the viability of cardiomyocytes (Figure S5C). However, Fer‐1 failed to further improve the viability of cardiomyocytes during valsartan treatment (Figure S5C). Mst1 knockdown abolished the protective role of valsartan (Figure S5C).

With valsartan treatment, Fer‐1 failed to further alleviate the H/R‐induced increase in lipid ROS, lipid peroxidation, and Fe^2+^ release (Figure S5D–F). Mst1 knockdown abrogated the inhibitory effect of valsartan on the H/R‐induced increase in lipid ROS, lipid peroxidation, and Fe^2+^ release (Figure S5D–F).

Following valsartan treatment, Fer‐1 failed to further improve the H/R‐induced decrease in GPX4, GSH, and NAPDH expression (Figure S5G–I). Mst1 knockdown abrogated the ability of valsartan to rescue GPX4, GSH, and NAPDH expression (Figure S5G–I). The IF analysis of RIPK3 and MLKL showed that there were no significant differences in the IF intensity of RIPK3 and MLKL between the H/R + valsartan group and the H/R + valsartan + Ad‐sh‐Mst1 group (Figure S5J,K). There was also no statistical difference in the membrane IF/cytoplasm IF, suggesting that the effect of Mst1 on MLKL activation was marginal (Figure S5J,K).

### 3.5 Valsartan Mitigated Myocardial IRI by Phosphorylating Mst1 at Thr183 to Inhibit Myocardial Ferritinophagy and Ferroptosis

Mst1 conditional knockout mice were generated and bred to establish a myocardial IRI model (Figure S6A–C). The effectiveness of Mst1 specific knockout was verified by the WB and IF assays (Figure S6D,E).

**FIGURE 6:**
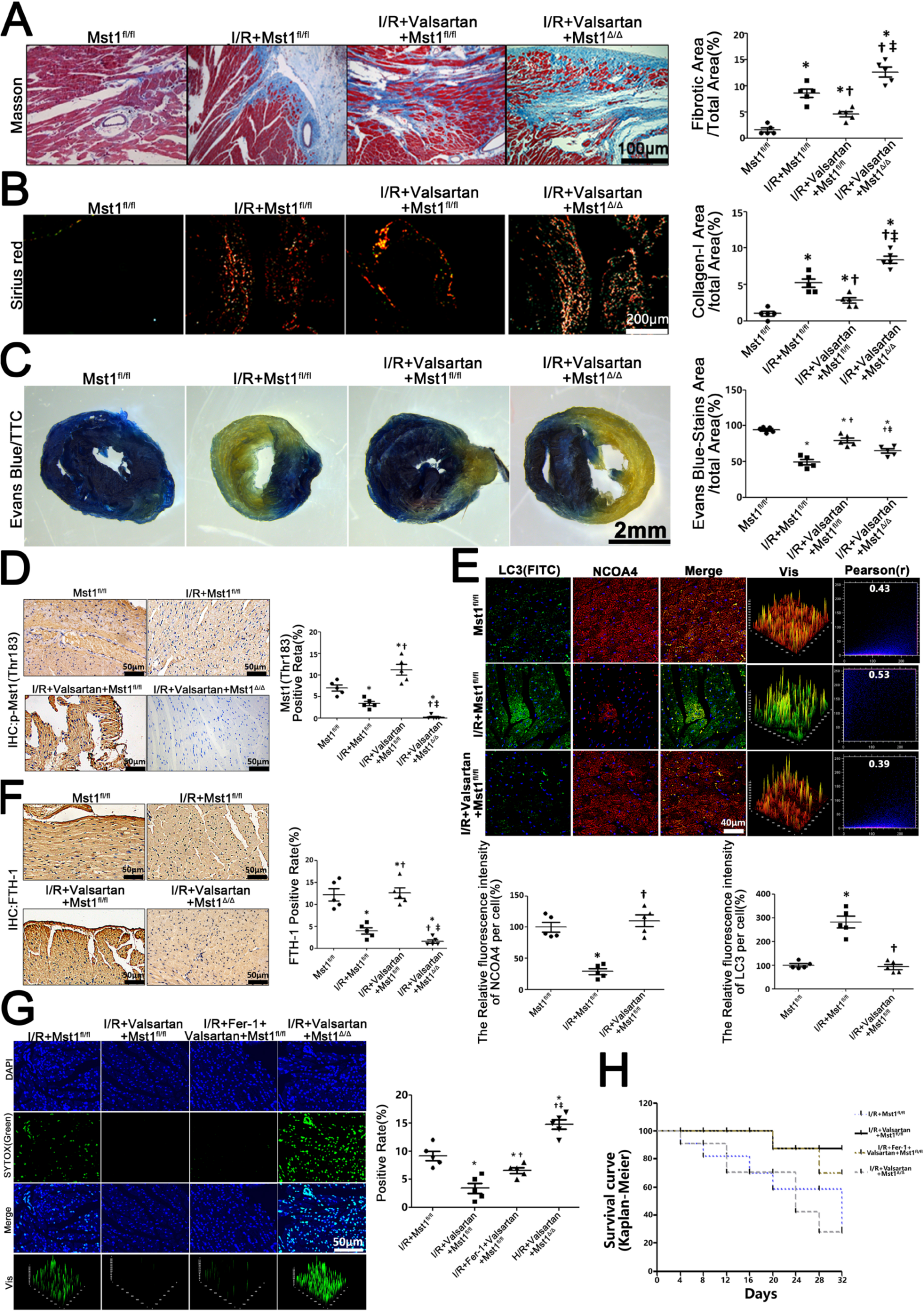
Valsartan mitigates myocardial IRI by phosphorylating Mst1 at Thr183 to inhibit myocardial ferritinophagy and ferroptosis. (A–G) The representative images of Masson trichrome, Sirius red and Evans Blue/TTC stains; The representative immunohistochemical images of *p*‐Mst1 (Thr183); The representative images of immunofluorescence co‐localisation between LC3 (FITC) and NCOA4 (CY3); The representative immunohistochemical images of FTH‐1; The representative immunofluorescence images of SYTOX (Green). Histogram: The fibrotic area/Total area%; Collagen‐I Area/Total area%; Evans Blue‐Stains Area/Total area%; Mst1 (Thr183) positive rate (%); Relative fluorescence intensity of NCOA4 and LC3 per field (Mst1^fl/fl^ group as 100%); FTH‐1 positive rate (%); *n* = 5. H: The survival analysis (Kaplan–Meier survival curve) in I/R (Mst1^fl/fl^), I/R + varsartan (Mst1^fl/fl^), I/R + Fer‐1 + varsartan (Mst1^fl/fl^), and I/R + varsartan (Mst1^Δ/Λ^). *p < 0.05 vs Mst1^fl/fl^ group, ^†^ *p* < 0.05 vs I/R+Mst1^fl/fl^ group, ^‡^ *p* < 0.05 vs I/R+Valsartan+Mst1^fl/fl^ group, *p < 0.05 vs I/R (Mst1^fl/fl^) group, ^†^ *p* < 0.05 vs I/R+Valsartan (Mst1^fl/fl^) group, ^‡^ *p* < 0.05 vs I/R+Fer‐1+Valsartan (Mst1^fl/fl^) group, (*n* = 5).

Masson's staining, Sirius red staining (polarised light), and Evans blue/TTC double staining demonstrated that valsartan mitigated myocardial fibrosis and diminished the infarct size induced by IRI (Figure 6A–C). In cardiomyocyte‐specific knockout Mst1 mice, the protective effect of valsartan on myocardial IRI was nullified (Figure 6A–C).

IHC and IF analyses revealed that valsartan effectively reversed the phosphorylation of Mst1 at Thr183, the reduction in ferritin and NCOA4 expression, and the upregulation of LC3 expression elicited by myocardial IRI. In cardiomyocyte‐specific Mst1 knockout mice, these effects of valsartan were notably absent (Figure 6D–F).

The SYTOX staining demonstrated that valsartan diminished the cardiomyocyte death induced by I/R. The concurrent administration of a ferroptosis inhibitor with valsartan did not yield additional amelioration of cardiomyocyte death, indicating that valsartan mitigates cardiomyocyte death by inhibiting ferroptosis. In cardiomyocyte‐specific Mst1 knockout mice, valsartan failed to ameliorate cardiomyocyte death induced by myocardial IRI (Figure 6G). Kaplan–Meier survival analysis showed that valsartan improved the survival rate of mice with myocardial IRI (Figure 6H). Notably, following valsartan treatment, Fer‐1 failed to further improve the survival rate of mice, and cardiomyocyte‐specific knockout Mst1 abrogated the protective effect of valsartan on mice subjected to myocardial I/R (Figure 6H).

**FIGURE 7:**
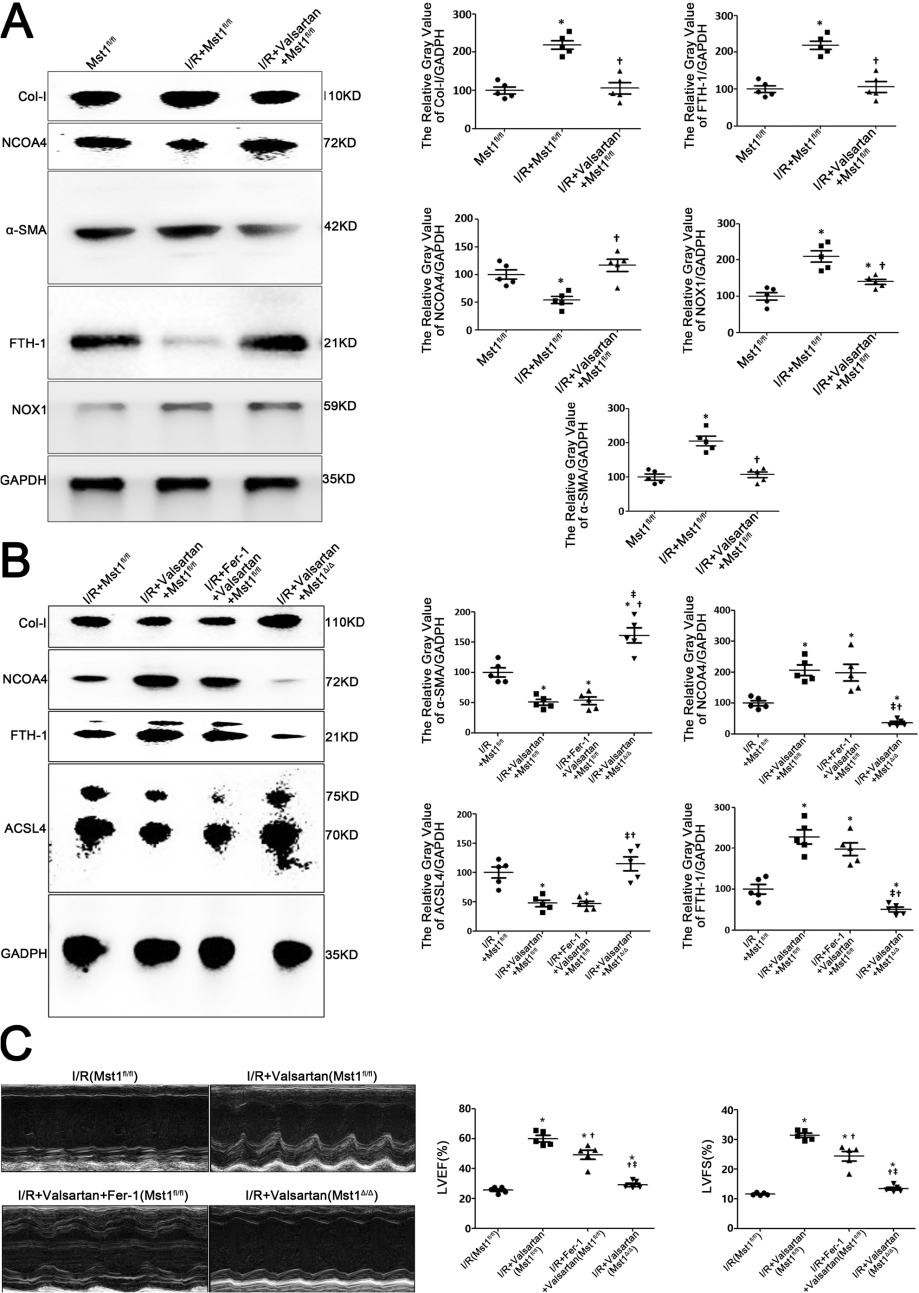
Valsartan mitigates myocardial IRI by phosphorylating Mst1 at Thr183 to inhibit myocardial ferritinophagy and ferroptosis. (A, B) Immunoblots and quantitative analyses of Col‐I/GAPDH, α‐SMA/GAPDH, NOX1/GAPDH, ACSL4/GAPDH, FTH‐1/GAPDH, and NCOA4/GAPDH. Histogram: The relative Grey Value of Col‐I/GAPDH, α‐SMA/GAPDH, NOX1/GAPDH, ACSL4/GAPDH, FTH‐1/GAPDH and NCOA4/GAPDH. *p < 0.05 vs. Mst1^fl/fl^ group; ^†^ *p* < 0.05 vs. I/R + Mst1^fl/fl^ group; *p < 0.05 vs. I/R + Mst1^fl/fl^ group; ^†^ *p* < 0.05 vs. I/R + Valsartan + Mst1^fl/fl^ group; ^‡^ *p* < 0.05 vs. I/R + Fer‐1 + Valsartan + Mst1^fl/fl^ group, (*n* = 5). (C) The representative images and histogram of echocardiography. Histogram: The LVEF (%) and LVFS (%). *p < 0.05 vs. I/R (Mst1^fl/fl^) group; ^†^ *p* < 0.05 vs. I/R + Valsartan (Mst1^fl/fl^) group; ^‡^ *p* < 0.05 vs. I/R + Fer‐1 + Valsartan (Mst1^fl/fl^) group, (*n* = 5).

WB assays demonstrated that valsartan mitigated myocardial fibrosis and ferroptosis induced by I/R, as evidenced by notable reductions in Col‐1, α‐SMA, and NOX1 expression (Figure 7A). I/R resulted in a decrease in myocardial ferritin and NCOA4 expression, which was counteracted by valsartan administration (Figure 7A). However, no significant alterations in the mRNA levels of FTH1 and NCOA4 were detected (Figure S7A,B).

Co‐IP assays revealed that I/R induced stronger interactions between NCOA4 and FTH1, which were reversed by valsartan (Figure S7C). WB experiments demonstrated that the incorporation of Fer‐1 into valsartan treatment failed to further ameliorate myocardial fibrosis and ferroptosis, as illustrated by the inability to reduce the expression levels of Col‐1, α‐SMA, and ACSL4 (Figure 7B). Furthermore, administration of Fer‐1 in combination with valsartan failed to result in any additional increases in myocardial ferritin and NCOA4 expression (Figure 7B). Valsartan counteracted an increase in myocardial MDA induced by I/R (Figure S7D). Furthermore, I/R increased the concentration of serum MDA, CK‐MB, LDH, and AST, and these effects were reversed by valsartan treatment (Figure S7E–H). The protective effect of valsartan was abolished by cardiomyocyte‐specific Mst1 knockout (Figure S7E–H).

Echocardiographic analysis demonstrated that valsartan improved cardiac function compromised by I/R (Figures 7C and S7I,J). Nonetheless, the concomitant administration of Fer‐1 with valsartan did not yield further enhancement of cardiac function, suggesting that valsartan ameliorates myocardial IRI by inhibiting myocardial ferroptosis (Figures 7C and S7I,J). In cardiomyocyte‐specific Mst1 knockout mice, valsartan failed to ameliorate the deterioration of cardiac function induced by I/R (Figures 7C and S7I,J).

In myocardium, valsartan mitigated the I/R‐induced increase of collagen fibre. However, the protective effect of valsartan was abolished by Mst1 specific knockout (Figure S7K). During this process, the mRNA levels of NCOA4 and FTH‐1 remained unchanged (Figure S8A,B). Following treatment with valsartan, ferroptosis inhibitors not only failed to further decrease the myocardial MDA content but also could not reduce the concentration of serum MDA, CK‐MB, LDH, and AST (Figure S8C–G).

DHE probing revealed that valsartan decreased the I/R‐induced production of ·O_2_^−^. However, upon treatment with valsartan, Fer‐1 failed to reduce the production of ·O_2_^−^ (Figure S8H). Mst1‐specific knockout abolished the ability of valsartan in ·O_2_^−^ suppression (Figure S8H).

In mice subjected to I/R, valsartan decreased the generation of myocardial ROS, Fe^2+^ and 4‐HNE, which was abrogated by the cardiomyocyte specific knockout Mst1 (Figure S8I–K). However, following treatment with valsartan, Fer‐1 failed to decrease the generation of myocardial ROS, Fe^2+^ and 4‐HNE (Figure S8I–K).

### 3.6 Administration of Valsartan Alleviated Myocardial IRI Caused by Revascularisation

**FIGURE 8:**
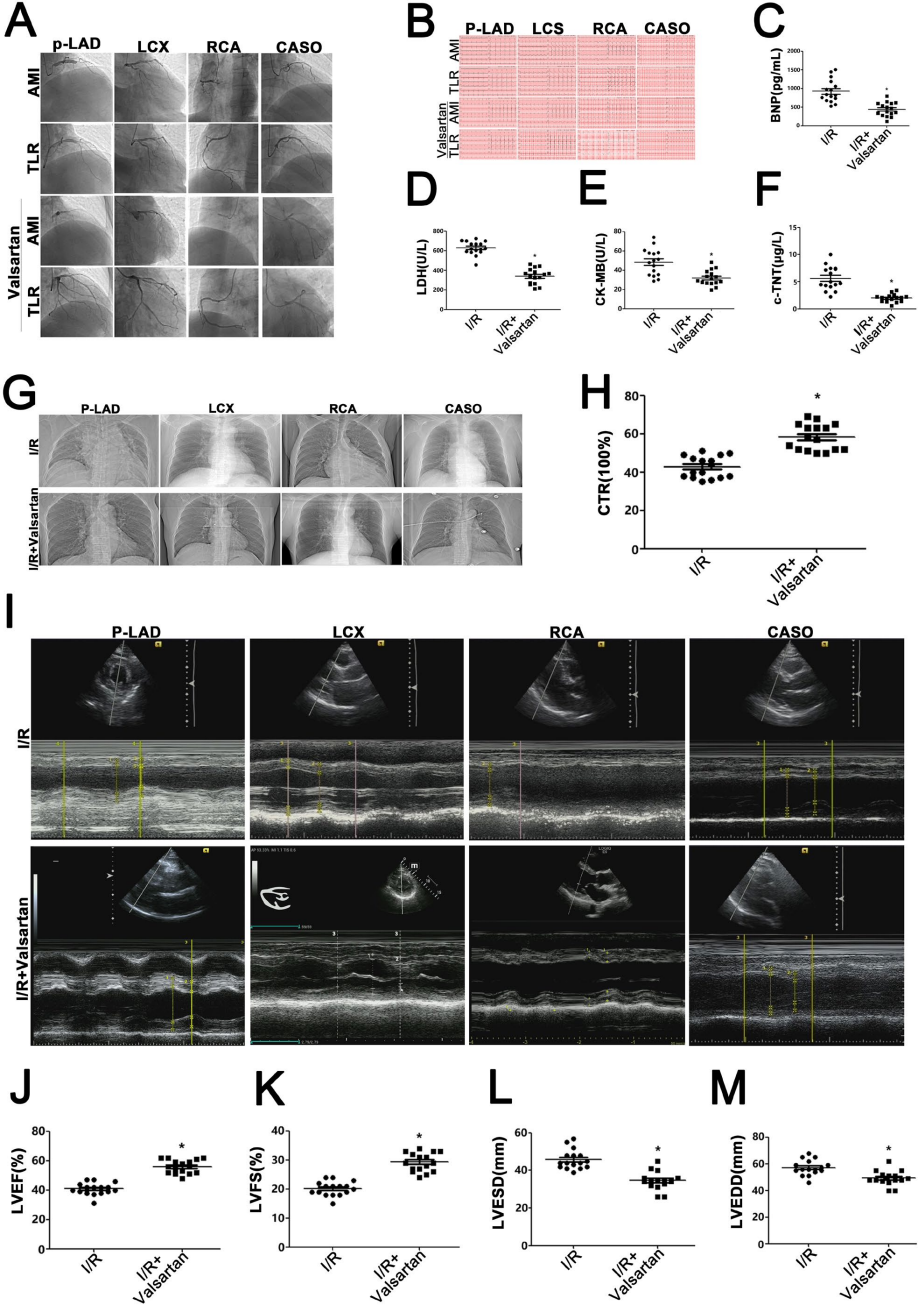
Administration of valsartan alleviates myocardial IRI caused by revascularisation. (A, B) The representative images of preoperative electrocardiogram and intraoperative coronary angiography (CAG). (C–F) The histogram of BNP, LDH, CK‐MB, and c‐TNT in patients post revascularisation. *p < 0.05 vs. I/R group, *n* = 16. (G, H) The representative images of Computed Tomography (CT) and cardiothoracic ratio (CTR %). *p < 0.05 vs. I/R group, *n* = 16. (I–M) The representative images and histogram of echocardiography. Histogram: The LVEF (%), LVFS (%), LVESD (mm) and LVEDD (mm). *p < 0.05 vs. I/R group, *n* = 16.

A total of 32 patients with AMI undergoing emergency revascularisation were enrolled in the present prospective, randomised, and controlled study. Patients were randomly assigned 1:1 to the valsartan pretreatment group (*n* = 16) and the conventional treatment group (*n* = 16), according to the clinical trial protocol (Figure 8A,B).

After revascularisation, cardiac function, cardiac morphology and serum levels of myocardial injury markers including BNP, LDH, CK‐MB and cTNT were systematically evaluated (Figure 8C–F). The results demonstrated that preoperative administration of valsartan effectively improved postoperative cardiac function, alleviated cardiac enlargement and significantly reduced serum concentrations of BNP, LDH, CK‐MB and cTNT compared with the control group (Figure 8C–F).

These clinical findings also confirm that valsartan pretreatment before revascularisation can inhibit heart enlargement and enhance cardiac function, suggesting an attenuation of revascularisation‐induced myocardial IRI and an improvement of cardiac function in patients with AMI (Figure 8G–M).

## 4 Discussion

Iron ions are involved in various enzymatic reactions in the body, such as DNA synthesis, mitochondrial oxidative phosphorylation for ATP production, erythrocyte maturation and the regulation of cell proliferation and organ development [28]. However, excessive free iron ions are highly reactive and induce the massive production of inflammatory factors and promote cellular ferroptosis through the generation of ROS [28, 29]. Recent studies have demonstrated that ferroptosis is closely associated with cardiovascular diseases [30]. For example, myocardial IRI is accompanied by ferroptosis, and its inhibition can significantly mitigate myocardial IRI [31].

Moderate ferritinophagy maintains the homeostasis of intracellular iron, whereas excessive ferritinophagy releases a large amount of free iron [7, 17]. This free iron catalyses the production of ROS via the Fenton reaction, leading to cellular damage through lipid peroxidation [7, 15, 17]. Myocardial ferroptosis and IRI, resulting from ferritinophagy in cardiomyocytes, is the leading cause of heart failure and sudden cardiac death [4]. This study also revealed that H/R and I/R treatment induced excessive ferritinophagy, ferroptosis, and mitochondrial damage in cardiomyocytes. Therefore, the regulation of ferritinophagy may offer a therapeutic strategy for myocardial ferroptosis [7, 17].

A crucial step in ferritinophagy involves the recruitment of ferritin [7, 18]. Iron ions bound to ferritin are subsequently released into the cytoplasm as free Fe^2+^, which triggers iron overload in cardiomyocytes and induces intracellular ROS generation [17, 18]. NCOA4 is a critical ferritinophagy receptor responsible for the recruitment of ferritin [7, 15, 17, 18]. Hou et al. reported that when ferritinophagy is excessive, NCOA4 turnover is promoted by the autophagy‐lysosome pathway, leading to a decrease in its protein expression but no change in its mRNA level [17, 32]. Notably, the present study revealed that H/R and I/R pretreatment decreased the protein expression of NCOA4, whereas the mRNA levels of NCOA4 remained unchanged, suggesting excessive ferritinophagy during myocardial I/R. Thus, ferritinophagy inhibitors are also under consideration to suppress myocardial IRI.

Our previous study demonstrated that the phosphorylation of Mst1 at Thr183 inhibits cardiomyocyte autophagy [14]. In the present study, Mst1 and Beclin1, an autophagy‐related molecule, were enriched in pathways associated with myocardial ferroptosis and IRI. There was also a positive correlation between Mst1 and Beclin1. Furthermore, the present study indicated that H/R and I/R treatment induce Mst1 (Thr183) dephosphorylation and Beclin1 (Ser295) dephosphorylation, leading to a decrease in the interaction between Beclin1 and Bcl‐2. Maejima et al. also reported that Mst1 induces an interaction between Beclin1 and Bcl‐2, and therefore inhibits cardiomyocyte autophagy by repressing the formation of ATG14L‐Beclin1, an autophagic initiation complex [33]. Sumneang et al. demonstrated that the dissociation of Beclin1 from the Beclin1‐Bcl‐2 complex inhibits the activity of the Xc transporter and impairs its ability to transport glutamate to the extracellular space and cystine into the cytoplasm, thereby exacerbating cardiomyocyte ferroptosis [8]. Taken together, it is suggested that the Mst1‐Beclin1 pathway may be involved in regulating ferritinophagy and ferroptosis during myocardial ferroptosis and I/R, and modulation of this pathway may represent a potential therapeutic strategy for myocardial ferroptosis and I/R.

In the present study, Spearman's correlation analysis indicated a positive correlation between AT_1_R and Mst1. Our previous study also revealed that Ang II inhibits Mst1 to increase autophagic flux in cardiomyocytes, which is dependent on AT_1_R (Angiotensin II Type 1 Receptor) [14]. Therefore, we hypothesised valsartan, a classic angiotensin II Type 1 receptor blockers (ARBs), may inhibit cardiomyocyte ferritinophagy and ferroptosis to alleviate myocardial IRI by regulating Mst1‐Beclin1 pathway. In proof of this, the present study revealed that valsartan restores the phosphorylation of Mst1 at Thr183 and that the valsartan‐induced phosphorylation of Mst1 restores the phosphorylation of Beclin1 at Ser295 to increase the interaction between Beclin1 and Bcl‐2, thus alleviating cardiomyocyte mitochondrial damage, inhibiting cardiomyocyte ferritinophagy and ferroptosis, and improving cardiomyocyte HRI in vitro and myocardial IRI in vivo.

Interestingly, this study reveals that the protective effects of valsartan against cardiomyocyte HRI and myocardial IRI are mediated by the inhibition of ferroptosis. Furthermore, valsartan alleviates myocardial IRI in an Mst1‐dependent manner, and the suppression of cardiomyocyte ferritinophagy and ferroptosis via the valsartan‐Mst1 axis is governed by AT_1_R. These findings further support our hypothesis that valsartan ameliorates myocardial ferroptosis and IRI by regulating the AT_1_R‐Mst1 pathway.

Autophagy inhibitors inhibit ferritinophagy [17]. However, several studies have reported that although autophagy inhibitors effectively reduce ferritinophagy in cardiomyocytes, they also inhibit mitophagy [34, 35, 36]. In cardiomyocytes, mitophagy results in the turnover of damaged mitochondria [34, 35, 36]. The inhibition of mitophagy by autophagy inhibitors results in the elimination of benefits from the inhibition of ferritinophagy and ferroptosis [34, 35, 36]. Therefore, exploring drugs other than classical autophagy inhibitors is important for improving myocardial IRI. Notably, the present study also revealed that valsartan inhibits ferritinophagy and ferroptosis while not disrupting mitophagy in cardiomyocytes, indicating that valsartan may break through the limitations of classic autophagy inhibitors, which involve impairment of mitophagy and result in mitochondrial damage.

In conclusion, H/R in vitro and I/R in vivo cause dephosphorylation of Mst1 at Thr183, and the dephosphorylation of Beclin1 at Ser295. This promotes the formation of the autophagy initiation complex, inducing ferritinophagy and ferroptosis in cardiomyocytes, which in turn triggers cardiomyocyte injury. Valsartan suppresses cardiomyocyte ferritinophagy and ferroptosis, and improves myocardial IRI by phosphorylating Mst1 at Thr183 to inhibit Beclin1, a crucial autophagy molecule. Administering valsartan before revascularisation may reduce myocardial IRI and improve the prognosis of patients with AMI. Figure S9 illustrates the mechanisms of the present study.

## 5 Limitations

The present study has several limitations that must be addressed. Firstly, owing to limited research funding, large‐scale clinical studies could not be carried out, and in the future, multicenter RCTs should be conducted. In addition, aside from valsartan, it remains to be seen whether other ARBs, such as olmesartan and telmisartan, could also improve myocardial IRI. Future studies should assess the effects of other ARBs on myocardial IRI.

## Author Contributions

**Xumin Wang:** writing – original draft, investigation, data curation, supervision, validation. **Yuanliang Sun:** investigation, software, visualization, validation. **Xin Li:** investigation, validation, visualization, formal analysis. **Kaikai Zhao:** resources, investigation, validation, software, methodology. **Zheng Cheng:** writing – original draft, writing – review and editing, funding acquisition, supervision, project administration, validation, visualization, resources, data curation, methodology, conceptualization. **Bixue Zhang:** conceptualization, methodology, data curation, supervision, validation. **Xintao Zhou:** resources, data curation, investigation, formal analysis. **Yuhong Wu:** resources, project administration, methodology, funding acquisition. **Qianqian Xu:** project administration, validation, software, visualization.

## Funding

The study was supported by the National Natural Science Foundation of China (grant no. 82070282), The China Postdoctoral Science Foundation (grant no. 2022MD723777), The General Projects of Key Research and Development Plan in Shaanxi Province (grant no. 2023‐YBSF‐346) and The Tianchi Talent Program (3rd) of Xinjiang Uygur Autonomous Region: Young Doctoral Talent.

## Ethics Statement

The clinical study was registered in the Chinese Clinical Trial Registry (TRN ChiCTR2100043501) and approved by The Medical Ethics Committee of Sinopharm Dongfeng General Hospital in Shiyan City of China (approval no. 2020016). All subjects signed written informed consent before participating in the study, and all personal information was strictly confidential. The experimental protocol of the animal study was approved by The Experimental Animal Ethics Committee of Xinjiang Medical University in Shiyan City of China (approval no. IACUC‐20250306‐19). All animal feeding, experimental operation, anaesthesia and euthanasia were carried out in strict accordance with the ethical review report and the guidelines of the Animal Care and Use Facility of Xinjiang Medical University.

## Consent

The authors have nothing to report.

## Conflicts of Interest

The authors declare no conflicts of interest.

## Supporting information


**Figure S1:** Autophagy and ferroptosis are associated with myocardial IRI and may involve Mst1 and Ang II. (A and B) The analyses of GESA (GSE43974), the enrichment plot and GESA results of pathways and biological function associated with Mst1. (C–E) Spearman correlation analysis between Mst1 and other genes (GSE43974).

**Figure S2:** H/R induces cardiomyocyte ferroptosis accompanied by Mst1 dephosphorylation at Thr183 and ferritinophagy overactivation. (A–K) The histogram of relative NCOA4 and FTH‐1 mRNA level; the Histogram of fold of ROS, LDH, LPO, Fe^2+^ release and NAPDH content; the MDA level, NAPDH oxidase activity, GPX4 activity. *p < 0.05 vs. Control group (Control group as 100%), *N* = 5.

**Figure S3:** Valsartan attenuates H/R‐induced cardiomyocyte ferroptosis and ferritinophagy by phosphorylating Mst1 at Thr183. (A and B) The histogram of relative NCOA4 and FTH‐1 mRNA level, Control group as 100%, *N* = 5. (C–E) The representative images of immunofluorescence co‐localisation between GFP‐LC3 and HBmTur‐Mito; Histogram: The relative immunofluorescence intensity of GFP‐LC3, The overlap rate of yellow/Green immunofluorescence (%). *p < 0.05 vs. Control group, ^†^ *p* < 0.05 vs. FCCP group, ^‡^ *p* < 0.05 vs. FCCP + 3‐MA group, ^§^ *p* < 0.05 vs. FCCP + CQ group, *N* = 4. (F) The viability of cardiomyocyte. Histogram: The OD value of Methylthiazolyldiphenyl‐tetrazolium bromide (MTT) assay. *p < 0.05 vs. Control group, ^†^ *p* < 0.05 vs. H/R group, *N* = 5. (G–L) The histogram of fold of ROS production, GSH content, NAPDH content and Fe^2+^ release, the MDA levels; the GPX4 activity. *p < 0.05 vs. Control group (Control group as 100%), ^†^ *p* < 0.05 vs. H/R group. *N* = 5.

**Figure S4:** Valsartan attenuates H/R‐induced cardiomyocytes ferroptosis by phosphorylating Mst1 to regulate ferritinophagy. (A–F) The representative images of immunofluorescence co‐localisation between FTH1 (FITC) and lysotracker (Red); between NCOA4 (FITC) and lysotracker (Red). Histogram: The overlap rate of Green/Red (%). The histogram of relative immunofluorescence intensity of FTH‐1 (FITC), lysotracker (Red) and NCOA4 (FITC). *p < 0.05 vs. H/R group (H/R group as 100%), *N* = 6. (G and H) The relative NCOA4 and FTH‐1 mRNA level, H/R group as 100%, *N* = 5.

**Figure S5:** Valsartan attenuates H/R‐induced cardiomyocytes ferroptosis by phosphorylating Mst1 to regulate ferritinophagy. (A and B) The relative NCOA4 and FTH‐1 mRNA level, H/R group as 100%, *N* = 5. (C) The representative images of cardiomyocyte morphology and the viability of cardiomyocyte; Histogram: The OD value of MTT assay. *p < 0.05 vs. H/R group, ^†^ *p* < 0.05 vs. H/R + valsartan group, ^‡^ *p* < 0.05 vs. H/R + Fer‐1 + valsartan group, *N* = 5. (D–I) The histogram of fold of ROS production, GSH content, NAPDH content and Fe^2+^ release, the MDA levels; the GPX4 activity. *p < 0.05 vs. H/R group (H/R group as 100%), ^†^ *p* < 0.05 vs. H/R + valsartan group, ^‡^ *p* < 0.05 vs. H/R + Fer‐1 + valsartan group, *N* = 5. (J and K) The representative images and histogram of fluorescent expression of RIPK3 (FITC) and MLKL (CY3), *N* = 5.

**Figure S6:** The construction of Mst1 conditional knockout mice. (A–C) The conditional knockout region, the constructed vector, the targeted allele, the conditional knockout allele, the constitutive knockout allele (after Cre recombination) and the sequence of the final targeting vector. (D) The representative images of Immunoblots. Histogram: The relative grey value of Mst1/Cofilin‐2 (%). Mst1^fl/fl^ group as 100%. *p < 0.05 vs. Mst1^fl/fl^ group, *N* = 3. (E) Representative immunofluorescence images of Mst1 (FITC); Histogram: The relative fluorescence intensity of Mst1 (FITC) per field (Mst1^fl/fl^ group as 100%), *p < 0.05 vs. Mst1^fl/fl^ group, *N* = 3.

**Figure S7:** Valsartan mitigates myocardial IRI by phosphorylating Mst1 at Thr183 to inhibit myocardial ferritinophagy and ferroptosis. (A and B) The relative NCOA4 and FTH‐1 mRNA level, Mst1^flox/flox^ group as 100%, *N* = 5. (C) The representative images of Co‐IP (IP: NCOA4). Histogram: The relative intensity of FTH‐1/NCOA4 (%), *p < 0.05 vs. Mst1^flox/flox^ group, ^†^ *p* < 0.05 vs. I/R + Mst1^flox/flox^ group. Mst1^flox/flox^ group as 100%, *N* = 4. (D–H) The histogram of myocardium MDA, serum MDA, serum CK‐MB, serum LDH and serum AST levels. *p < 0.05 vs. Mst1^flox/flox^ group, ^†^ *p* < 0.05 vs. I/R + Mst1^flox/flox^ group, ^‡^ *p* < 0.05 vs. I/R + valsartan + Mst1^flox/flox^ group, *N* = 5. (I and J) The histogram of LVEF and LVFS in echocardiography of Mst1^flox/flox^ and Mst1^Λ/Λ^ mice. *p < 0.05 vs. Mst1^flox/flox^ group, ^†^ *p* < 0.05 vs. I/R + Mst1^flox/flox^ group, ^‡^ *p* < 0.05 vs. I/R + valsartan + Mst1^flox/flox^ group, *N* = 5. (K) The polarised light intensity of Sirius Red Staining.

**Figure S8:** Valsartan mitigates myocardial IRI by phosphorylating Mst1 at Thr183 to inhibit myocardial ferritinophagy and ferroptosis. (A and B) The relative NCOA4 and FTH‐1 mRNA level, I/R + Mst1^flox/flox^ group as 100%, *N* = 5. (C–G) The myocardium MDA, serum MDA, serum CK‐MB, serum LDH and serum AST level. (H and I) The representative immunofluorescence images of DHE probe. Histogram: The relative fold of ROS. *p < 0.05 vs. Mst1^flox/flox^ group, ^†^ *p* < 0.05 vs. I/R + Mst1^flox/flox^ group, ^‡^ *p* < 0.05 vs. I/R + valsartan + Mst1^flox/flox^ group. I/R + Mst1^flox/flox^ group as 100%. I/R + Mst1^flox/flox^ group as 100%, *N* = 5. (J and K) Histogram: The relative fold of Fe^2+^ and 4‐HNE. *p < 0.05 vs. Mst1^flox/flox^ group, ^†^ *p* < 0.05 vs. I/R + Mst1^flox/flox^ group, ^‡^ *p* < 0.05 vs. I/R + valsartan + Mst1^flox/flox^ group. I/R + Mst1^flox/flox^ group as 100%, *N* = 5.

**Figure S9:** Schematic diagram of this article.

## Data Availability

The data (detailed experimental procedures, raw electrophoresis gels, etc.) that support the findings are available from the corresponding author. Detailed clinical information about patients is not publicly available due to patient privacy protection.
