# Supplementary figures and images for: Valsartan Reduces Myocardial Ischemia–Reperfusion Injury by Inhibiting Ferritinophagy‐Mediated Ferroptosis
Source: J Cell Mol Med. 2026 Jul 2;30(13):e71269. doi: 10.1111/jcmm.71269 (PMC13329122; doi:10.1111/jcmm.71269)

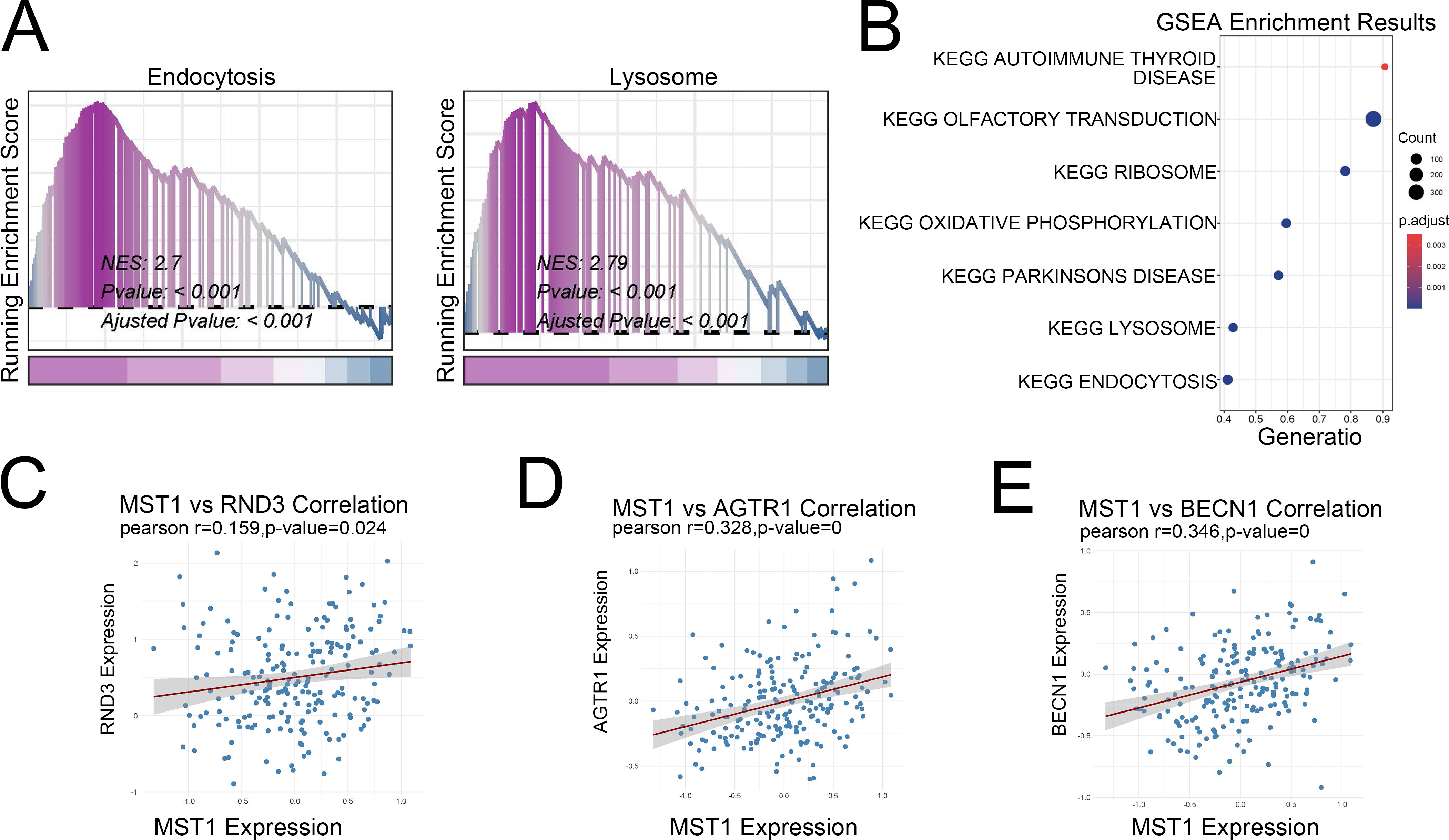

Supplement: Supplementary file 1 — Figure S1: Autophagy and ferroptosis are associated with myocardial IRI and may involve Mst1 and Ang II. (A and B) The analyses of GESA (GSE43974), the enrichment plot and GESA results of pathways and biological function associated with Mst1. (C–E) Spearman correlation analysis between Mst1 and other genes (GSE43974). [file JCMM-30-e71269-s009.jpg]

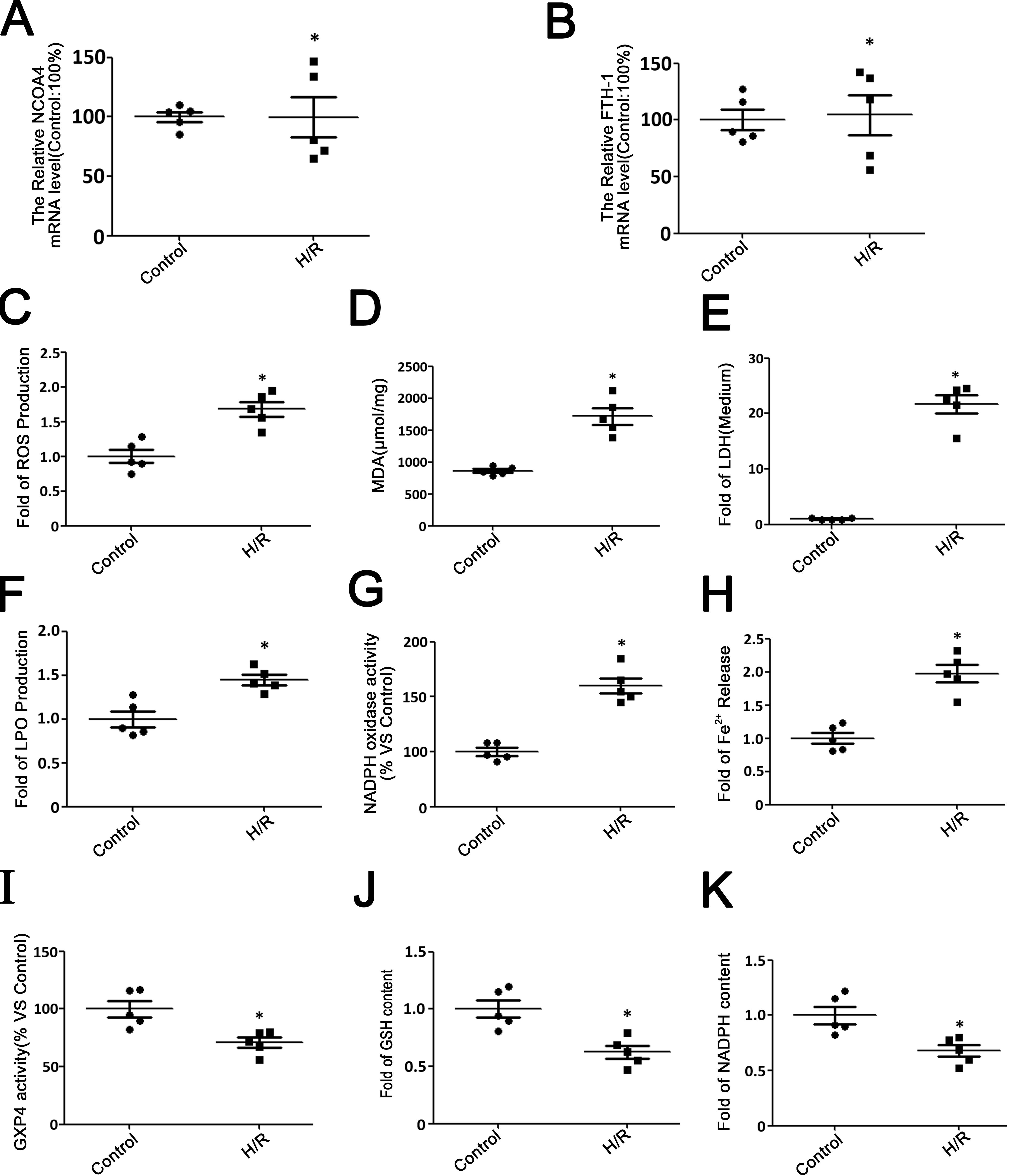

Supplement: Supplementary file 2 — Figure S2: H/R induces cardiomyocyte ferroptosis accompanied by Mst1 dephosphorylation at Thr183 and ferritinophagy overactivation. (A–K) The histogram of relative NCOA4 and FTH‐1 mRNA level; the Histogram of fold of ROS, LDH, LPO, Fe2+ release and NAPDH content; the MDA level, NAPDH oxidase activity, GPX4 activity. *p < 0.05 vs. Control group (Control group as 100%), N = 5. [file JCMM-30-e71269-s007.jpg]

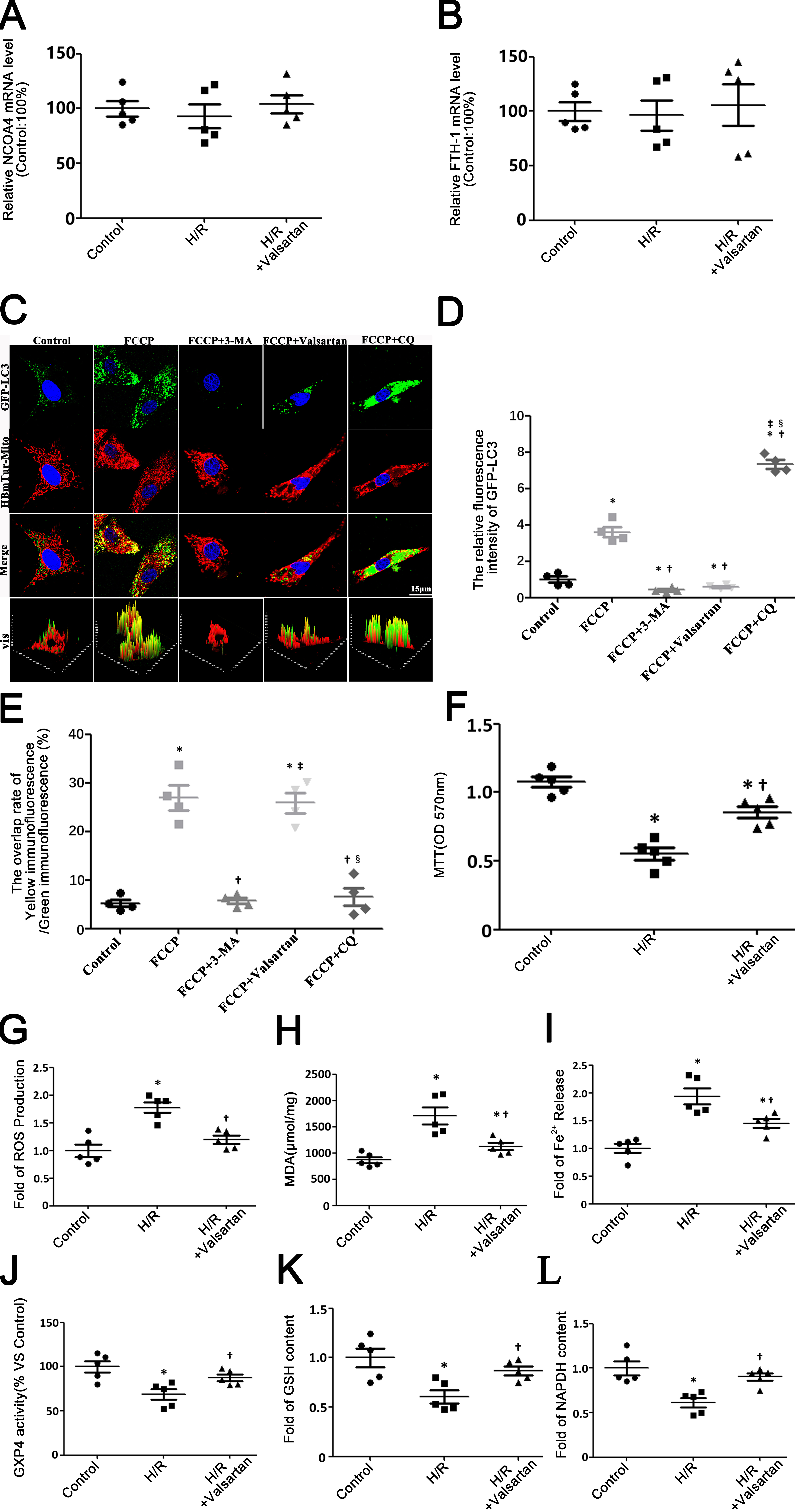

Supplement: Supplementary file 3 — Figure S3: Valsartan attenuates H/R‐induced cardiomyocyte ferroptosis and ferritinophagy by phosphorylating Mst1 at Thr183. (A and B) The histogram of relative NCOA4 and FTH‐1 mRNA level, Control group as 100%, N = 5. (C–E) The representative images of immunofluorescence co‐localisation between GFP‐LC3 and HBmTur‐Mito; Histogram: The relative immunofluorescence intensity of GFP‐LC3, The overlap rate of yellow/Green immunofluorescence (%). *p < 0.05 vs. Control group, † p < 0.05 vs. FCCP group, ‡ p < 0.05 vs. FCCP + 3‐MA group, § p < 0.05 vs. FCCP + CQ group, N = 4. (F) The viability of cardiomyocyte. Histogram: The OD value of Methylthiazolyldiphenyl‐tetrazolium bromide (MTT) assay. *p < 0.05 vs. Control group, † p < 0.05 vs. H/R group, N = 5. (G–L) The histogram of fold of ROS production, GSH content, NAPDH content and Fe2+ release, the MDA levels; the GPX4 activity. *p < 0.05 vs. Control group (Control group as 100%), † p < 0.05 vs. H/R group. N = 5. [file JCMM-30-e71269-s003.jpg]

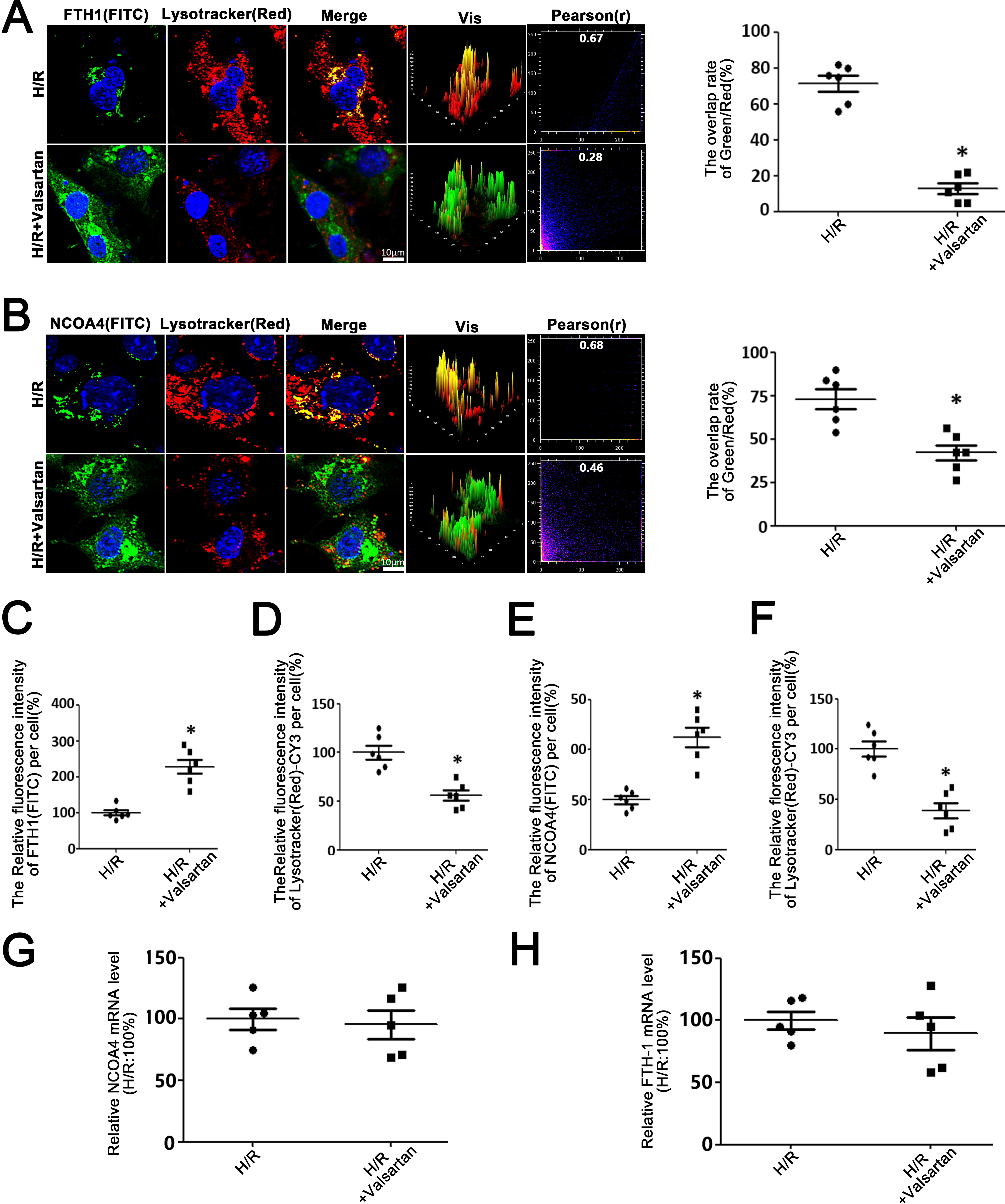

Supplement: Supplementary file 4 — Figure S4: Valsartan attenuates H/R‐induced cardiomyocytes ferroptosis by phosphorylating Mst1 to regulate ferritinophagy. (A–F) The representative images of immunofluorescence co‐localisation between FTH1 (FITC) and lysotracker (Red); between NCOA4 (FITC) and lysotracker (Red). Histogram: The overlap rate of Green/Red (%). The histogram of relative immunofluorescence intensity of FTH‐1 (FITC), lysotracker (Red) and NCOA4 (FITC). *p < 0.05 vs. H/R group (H/R group as 100%), N = 6. (G and H) The relative NCOA4 and FTH‐1 mRNA level, H/R group as 100%, N = 5. [file JCMM-30-e71269-s006.jpg]

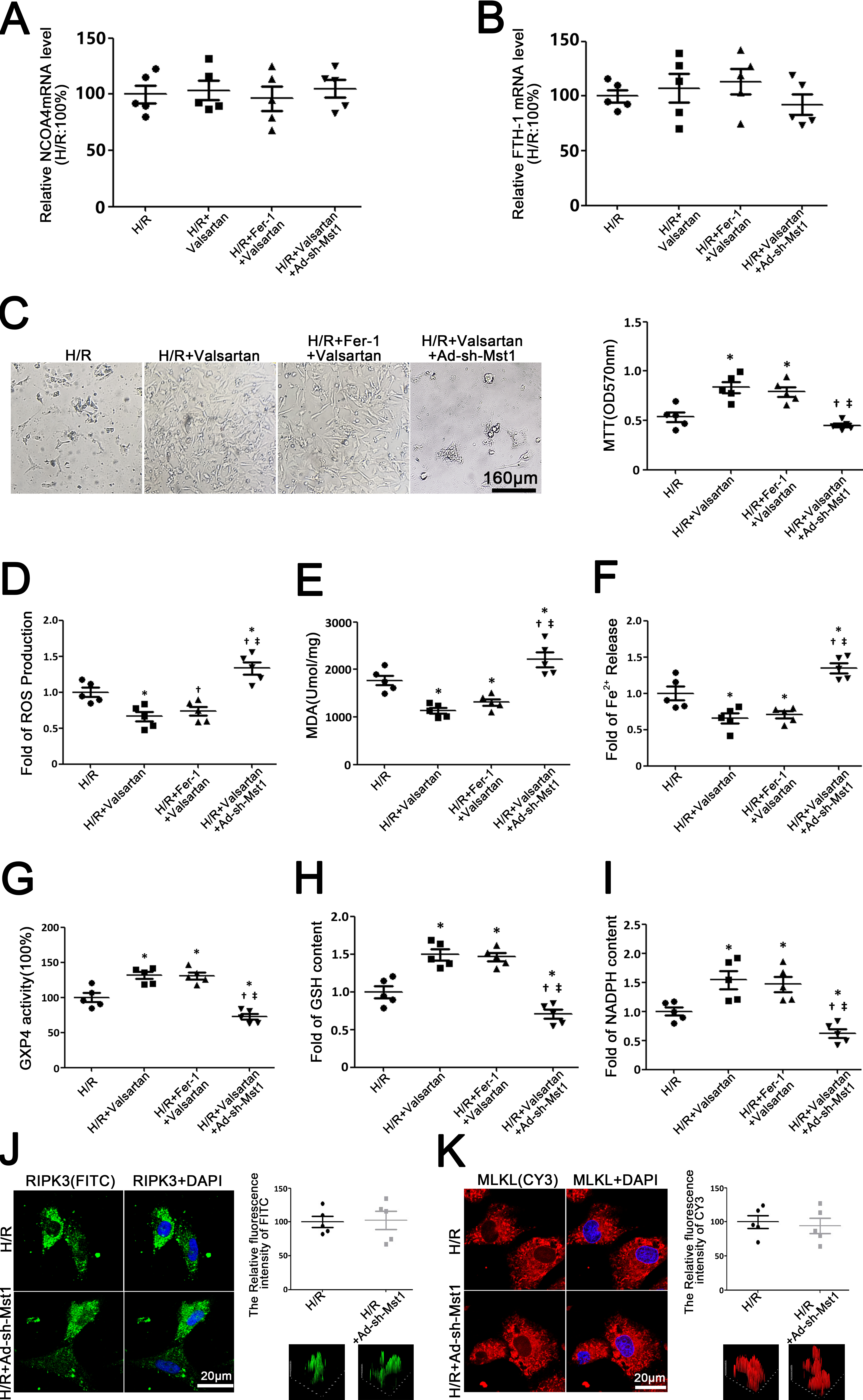

Supplement: Supplementary file 5 — Figure S5: Valsartan attenuates H/R‐induced cardiomyocytes ferroptosis by phosphorylating Mst1 to regulate ferritinophagy. (A and B) The relative NCOA4 and FTH‐1 mRNA level, H/R group as 100%, N = 5. (C) The representative images of cardiomyocyte morphology and the viability of cardiomyocyte; Histogram: The OD value of MTT assay. *p < 0.05 vs. H/R group, † p < 0.05 vs. H/R + valsartan group, ‡ p < 0.05 vs. H/R + Fer‐1 + valsartan group, N = 5. (D–I) The histogram of fold of ROS production, GSH content, NAPDH content and Fe2+ release, the MDA levels; the GPX4 activity. *p < 0.05 vs. H/R group (H/R group as 100%), † p < 0.05 vs. H/R + valsartan group, ‡ p < 0.05 vs. H/R + Fer‐1 + valsartan group, N = 5. (J and K) The representative images and histogram of fluorescent expression of RIPK3 (FITC) and MLKL (CY3), N = 5. [file JCMM-30-e71269-s008.jpg]

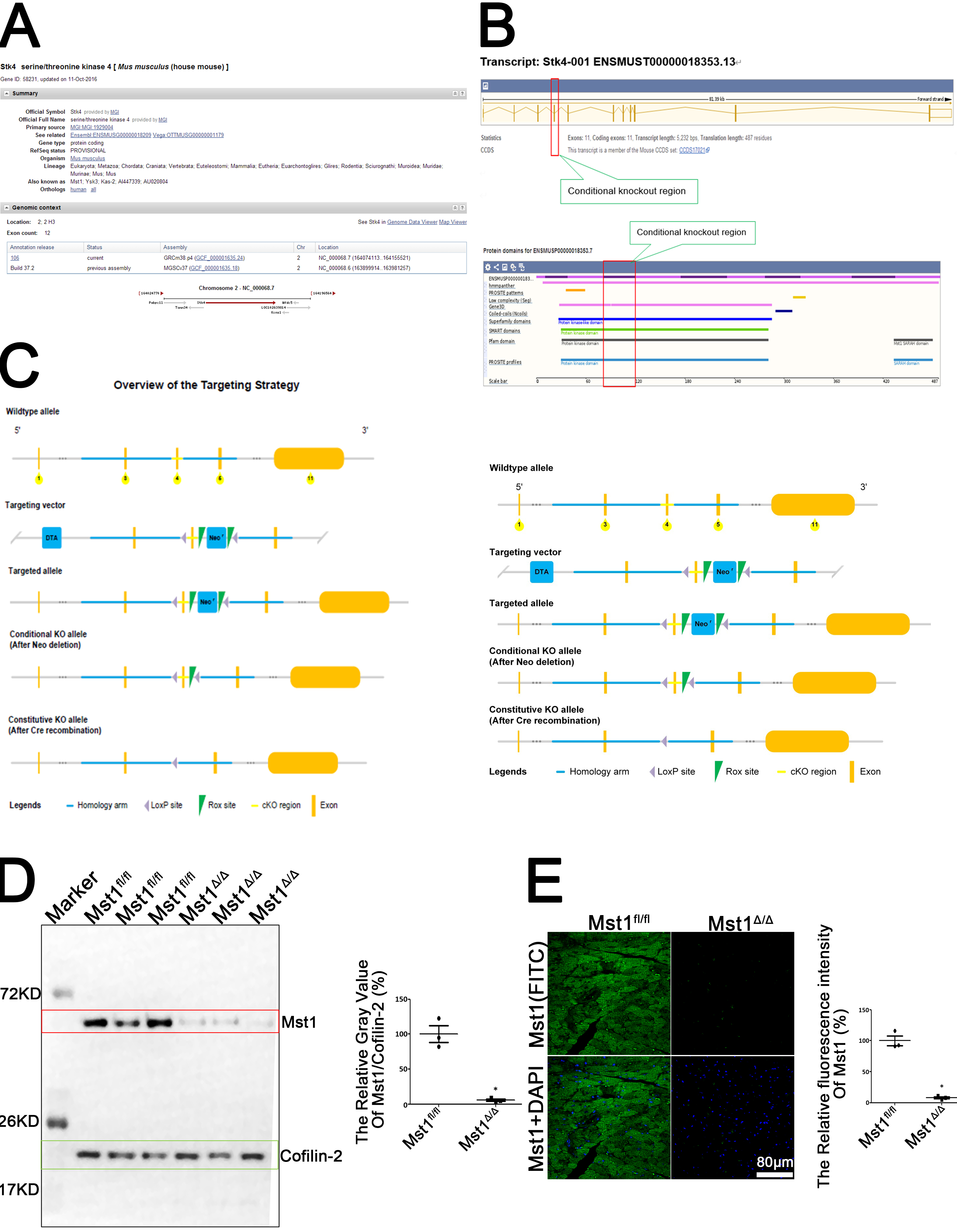

Supplement: Supplementary file 6 — Figure S6: The construction of Mst1 conditional knockout mice. (A–C) The conditional knockout region, the constructed vector, the targeted allele, the conditional knockout allele, the constitutive knockout allele (after Cre recombination) and the sequence of the final targeting vector. (D) The representative images of Immunoblots. Histogram: The relative grey value of Mst1/Cofilin‐2 (%). Mst1fl/fl group as 100%. *p < 0.05 vs. Mst1fl/fl group, N = 3. (E) Representative immunofluorescence images of Mst1 (FITC); Histogram: The relative fluorescence intensity of Mst1 (FITC) per field (Mst1fl/fl group as 100%), *p < 0.05 vs. Mst1fl/fl group, N = 3. [file JCMM-30-e71269-s001.jpg]

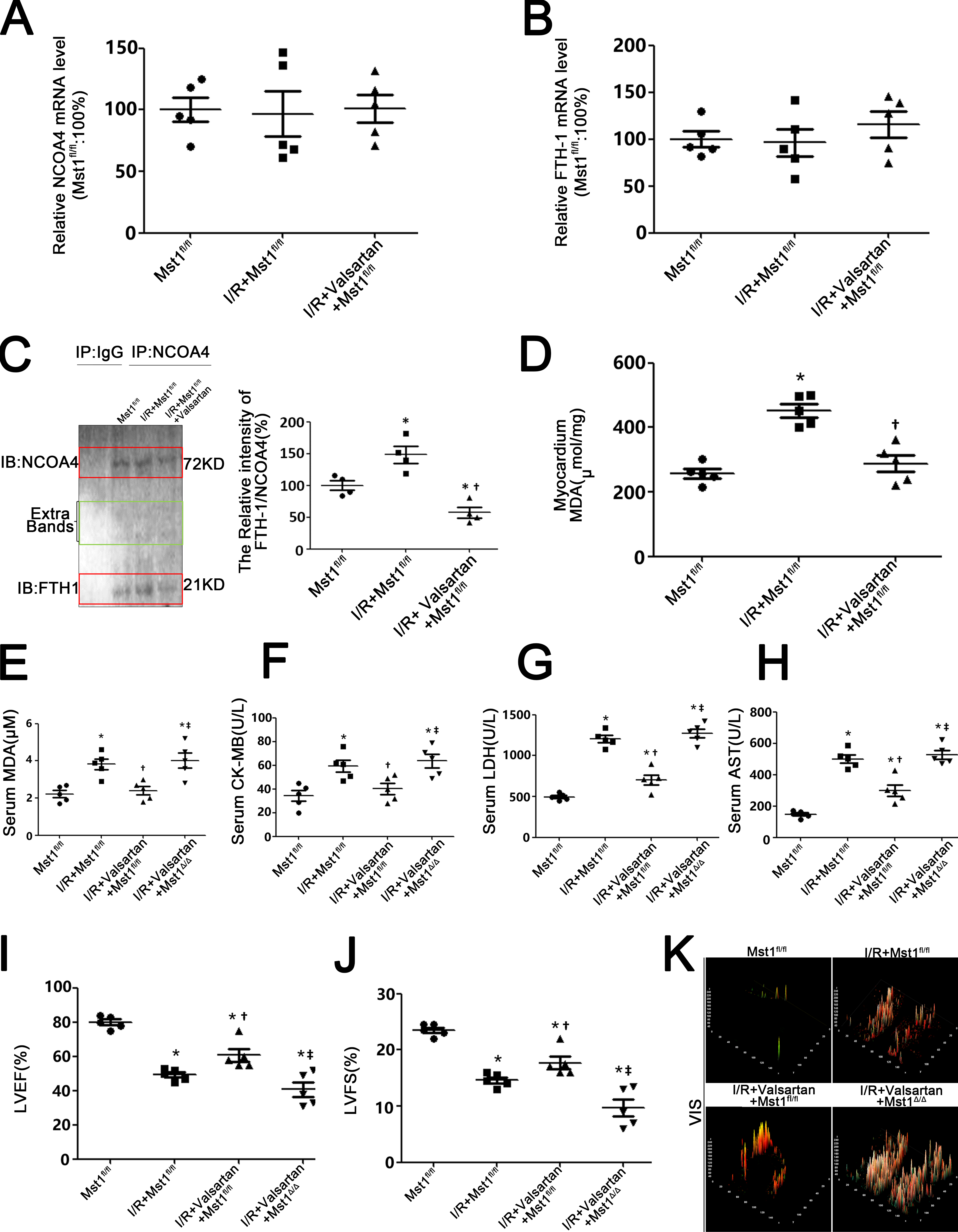

Supplement: Supplementary file 7 — Figure S7: Valsartan mitigates myocardial IRI by phosphorylating Mst1 at Thr183 to inhibit myocardial ferritinophagy and ferroptosis. (A and B) The relative NCOA4 and FTH‐1 mRNA level, Mst1flox/flox group as 100%, N = 5. (C) The representative images of Co‐IP (IP: NCOA4). Histogram: The relative intensity of FTH‐1/NCOA4 (%), *p < 0.05 vs. Mst1flox/flox group, † p < 0.05 vs. I/R + Mst1flox/flox group. Mst1flox/flox group as 100%, N = 4. (D–H) The histogram of myocardium MDA, serum MDA, serum CK‐MB, serum LDH and serum AST levels. *p < 0.05 vs. Mst1flox/flox group, † p < 0.05 vs. I/R + Mst1flox/flox group, ‡ p < 0.05 vs. I/R + valsartan + Mst1flox/flox group, N = 5. (I and J) The histogram of LVEF and LVFS in echocardiography of Mst1flox/flox and Mst1Λ/Λ mice. *p < 0.05 vs. Mst1flox/flox group, † p < 0.05 vs. I/R + Mst1flox/flox group, ‡ p < 0.05 vs. I/R + valsartan + Mst1flox/flox group, N = 5. (K) The polarised light intensity of Sirius Red Staining. [file JCMM-30-e71269-s005.jpg]

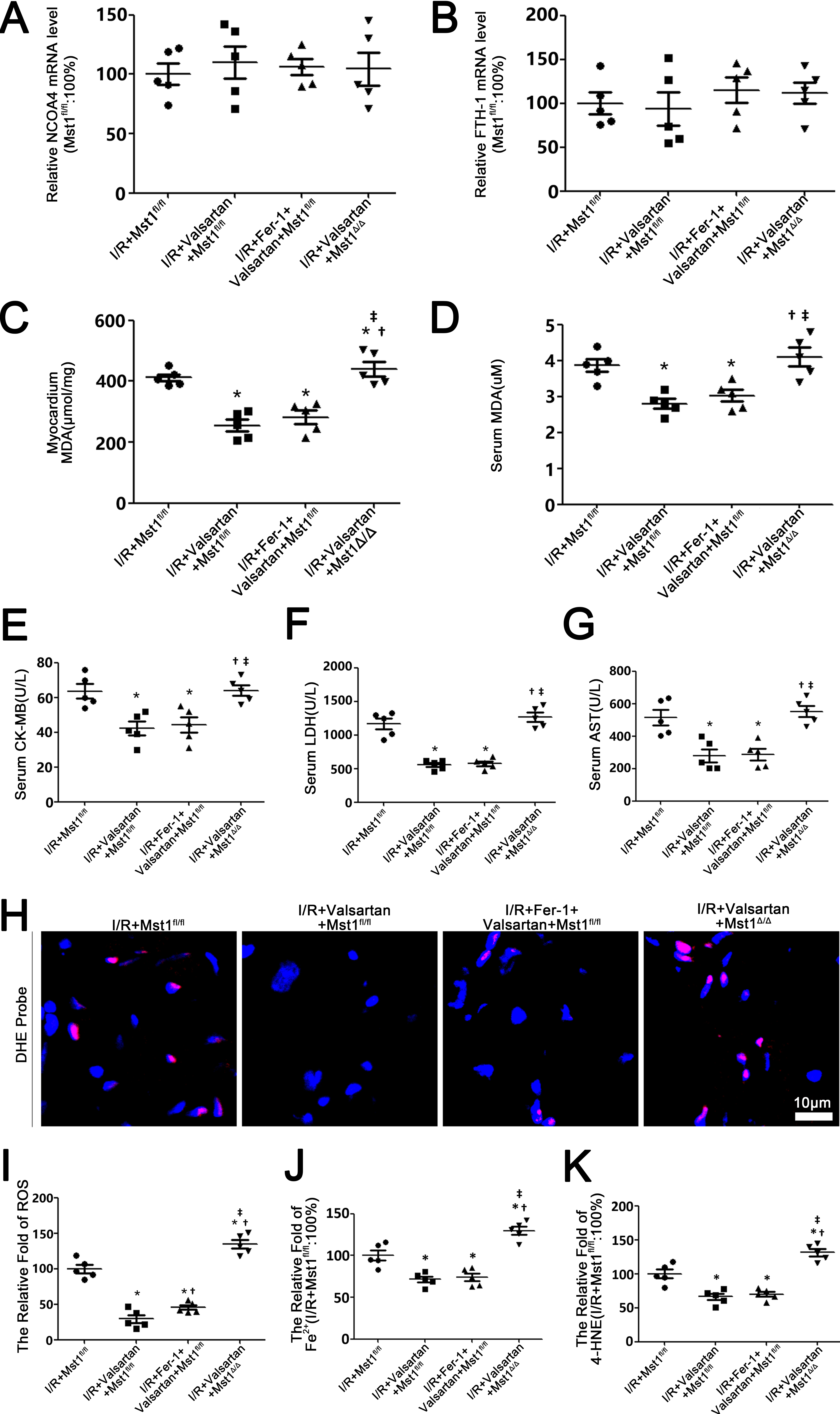

Supplement: Supplementary file 8 — Figure S8: Valsartan mitigates myocardial IRI by phosphorylating Mst1 at Thr183 to inhibit myocardial ferritinophagy and ferroptosis. (A and B) The relative NCOA4 and FTH‐1 mRNA level, I/R + Mst1flox/flox group as 100%, N = 5. (C–G) The myocardium MDA, serum MDA, serum CK‐MB, serum LDH and serum AST level. (H and I) The representative immunofluorescence images of DHE probe. Histogram: The relative fold of ROS. *p < 0.05 vs. Mst1flox/flox group, † p < 0.05 vs. I/R + Mst1flox/flox group, ‡ p < 0.05 vs. I/R + valsartan + Mst1flox/flox group. I/R + Mst1flox/flox group as 100%. I/R + Mst1flox/flox group as 100%, N = 5. (J and K) Histogram: The relative fold of Fe2+ and 4‐HNE. *p < 0.05 vs. Mst1flox/flox group, † p < 0.05 vs. I/R + Mst1flox/flox group, ‡ p < 0.05 vs. I/R + valsartan + Mst1flox/flox group. I/R + Mst1flox/flox group as 100%, N = 5. [file JCMM-30-e71269-s002.jpg]

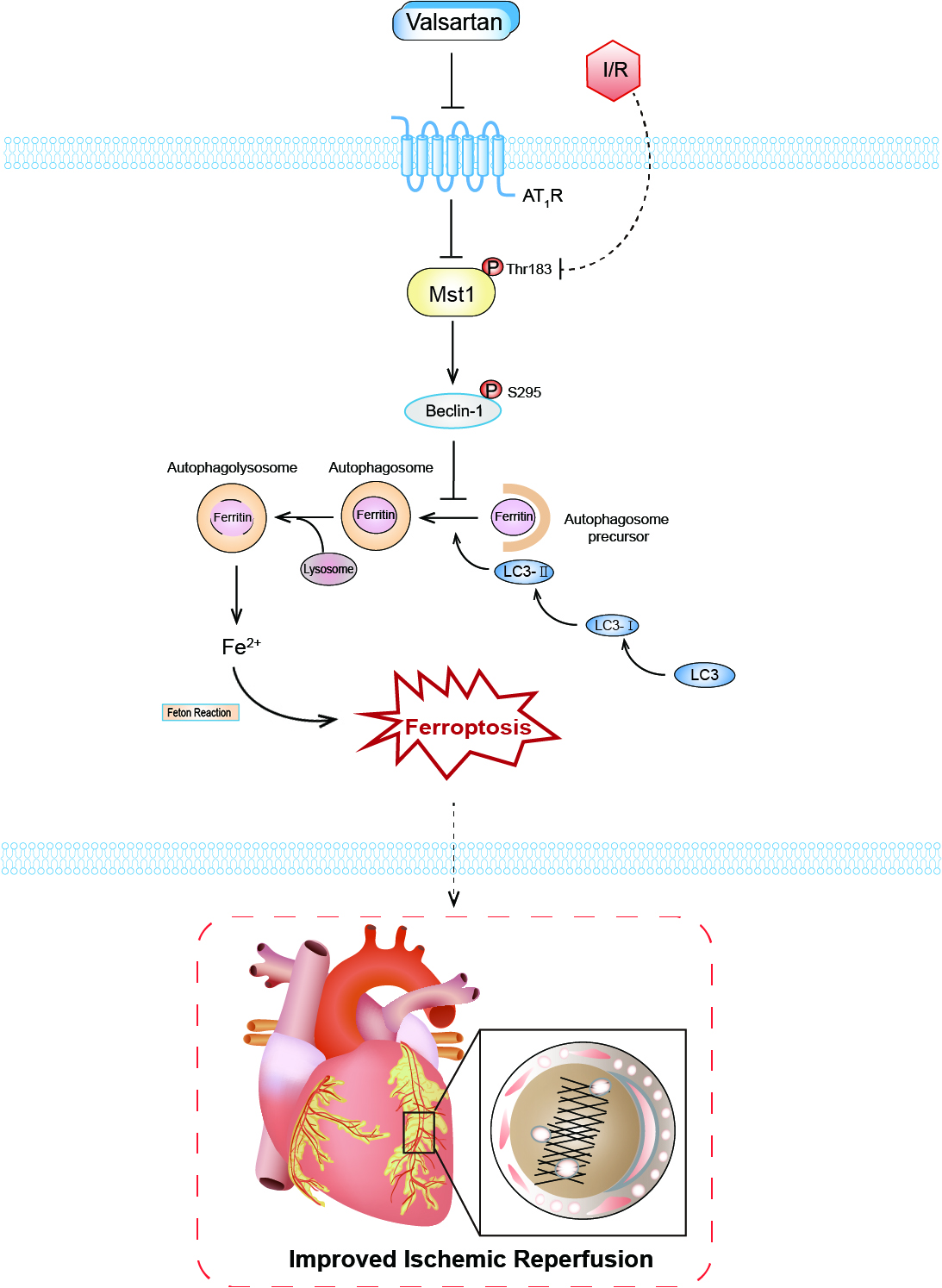

Supplement: Supplementary file 9 — Figure S9: Schematic diagram of this article. [file JCMM-30-e71269-s004.bmp]
